# Safety of intrauterine devices in MRI

**DOI:** 10.1371/journal.pone.0204220

**Published:** 2018-10-09

**Authors:** Simon Bussmann, Roger Luechinger, Johannes M. Froehlich, Constantin von Weymarn, Carolin Reischauer, Dow Mu Koh, Andreas Gutzeit

**Affiliations:** 1 Department of Chemistry and Applied Biosciences, ETH Zuerich, Zuerich, Switzerland; 2 Department of Radiology, Clinical Research unit of St. Anna Hospital Luzern, Lucerne, Switzerland; 3 Institute for Biomedical Engineering, University and ETH Zuerich, Zuerich, Switzerland; 4 Department of Radiology, Royal Marsden Hospital, Sutton, United Kingdom; 5 Department of Radiology, Paracelsus Medical University, Salzburg, Austria; Linköping University, SWEDEN

## Abstract

**Objectives:**

The paucity of safety information on intrauterine devices (IUD) for magnetic resonance imaging (MRI) examinations is clinically relevant. The aim of this study is to evaluate the MRI safety of clinically used IUDs composed of copper/gold and stainless steel at 1.5T and 3.0T.

**Materials and methods:**

We assessed and compared the displacement force, torque effects, presence of imaging artifacts and heating of IUDs composed of copper/gold (western IUDs) and stainless steel (China) on 1.5 and 3.0T MRI systems.

**Results:**

Gold/Copper IUDs can show small deflection angles of 7° ± 7° in the worst-case field gradient of 40T/m (equivalent to magnetic force of 0.5 mN), while the stainless steel IUD experienced significant magnetic force and deflection (Force > 7.5 N; deflection angle 90° ± 1°). Manual rotation and suspension method show no torque effects on gold/copper IUDs but high torque effects were observed by manual rotation on the stainless steel IUD. Heating measurements showed a temperature increase (rescaled to a wbSAR of 4 W/kg) of 1.4°C at 1.5T / 3.4°C at 3.0 T (stainless steel IUD), 3.2°C at 1.5 T / 3.8°C at 3 T (copper/gold IUD), 3.3°C at 1.5 T / 4.8°C at 3 T (copper 1), 3.8°C at 1.5 T / 4.8°C at 3 T (copper 2). The visible imaging artifacts of the copper and gold IUDs at 3 T MRI reach a diameter of 4 mm ± 1 mm, while the stainless steel IUD resulted in artifacts measuring 200mm ± 10 mm when using gradient echo pulse sequences.

**Conclusions:**

Standard IUDs (copper/gold) can be considered as conditional for MR safety at 1.5 T and 3.0 T, demonstrating at wbSAR up to 4W/kg and a magnetic field gradient of up to 40T/m with minimal imaging artifacts. The stainless steel IUD, however, induces unacceptable artifacts and is potentially harmful to patients during MRI due to high magnetic dislocation forces and torque (MR unsafe).

## Introduction

With the rising global population in recent decades, effective contraceptive methods which are safe, reliable and cheap are needed for family planning [1]. The intrauterine device (IUD), a contraceptive method that is locally deployed into the uterine cavity, has been shown to effectively prevent pregnancies [references?]. IUDs are of two general types, hormonal IUDs and metallic IUDs, the latter are usually made of copper or stainless steel. As IUDs are clinically effective and their effects reversible by removal, many women choose IUD to prevent pregnancies. In the United States of America and Europe, there is rising utilization of IUDs in about 2% and 6% of women respectively [2,3].

In Asia, especially in China, recent estimates show that 44% of all Chinese women between 15–45 years of age use IUDs. Until recently, there was a mandatory one-child policy in China, and the deployment of IUDs are therefore particular significant. Many different types of IUDs have been in use in China [4]. One product that was widely used in the 1980’s (more than 90% of all IUDs used in China at that time) is made of stainless steel, and known as the “Chinese ring” [4]. Although the production of this particular IUD was stopped in 2000, the. long lifespan of the Chinese ring, which is between 5 to 20 years, means that it may be left in-situ in some elder Chinese women [5]. To our knowledge, there is only one international publication, which describes this ring from a radiological point of view [6].

There is a paucity of publications that address the safety of IUDs during MR imaging. Most of these publications do not consider comprehensively measure relevant aspects such as heating, torque, magnetic force and artifacts on both 1.5T and 3T MRI systems. Furthermore, existing publications focus on copper IUDs and have not systematically addressed IUDs that contain gold or steel.[7, 8] Although IUDs are considered as clinically safe, we could not find a publication describing the safety of IUDs made of different materials at higher MR field stengths, especially during MRI investigations.

From a radiological point of view, the lack of safety information on a range of IUDs within a MRI magnet field, poses a relevant clinical problem for radiologists and gynecologists. There is an ongoing and sometimes controversial debate concerning whether every IUD is safe to undergo MRI investigation, whether IUD placement must be checked after scanning or whether these devices may be dangerous for women during MRI [8]. For our study we selected the Nova T 380, Mona Lisa Cu375 (identical to Multiload Cu 375), Gold Luna and the Chinese ring IUD for investigation. Hormonal IUDs have not been included in this study, since these do not include any metallic, magnetic, or conductive materials and therefore can even be labeled as MR safe on a scientific rationale.

To our knowledge, there are no prior published studies investigating the safety behavior of these devices including the Chinese ring; nor has there been a previous systematic investigation of the temperature change and degree of movement of these IUDs induced by magnetic fields on 1.5 T and 3.0 T MR systems.

Hence, the aim of this study was to investigate the safety of the most frequently used IUDs during MRI at 1.5 T and 3.0 T. This study hopes to clarify whether radiologists should apply IUD specific precaution measures in clinical practice and whether the IUD placement of copper/gold IUD needs to be re-verified after MRI examination.

## Materials and methods

The primary objective of the present study was to assess the safety of IUDs in an MRI environment. The following aspects were tested: (1) Assessment of the displacement force and torque effects of the implant in the main static magnetic field at 3.0 T MRI (following ASTM F2052-15 [9] and ASTM F2213-17 [10]). (2) Evaluation of heating effects due to the RF-field during MR scans at 1.5 T and 3 T (following ASTM F2182-11a [11]) and (3): Assessment of the size of image artifacts at 3.0 T (following ASTM F2119-07 (2013) [12]).

The MR measurements were undertaken between 01.01.2016 and 01.04.2016. Investigation of the IUDs were realized on the following MRI platforms: 1.5 T (Achieva, Philips Healthcare, Best, The Netherlands) for heating measurements; 3.0 T (Achieva, Philips Healthcare, Best, The Netherlands) for heating measurements, force and torque; 3.0 T (Ingenia, Philips Healthcare, Best, the Netherlands) for artifact measurements.

### IUDs evaluated

To our knowledge and own literature search, there are five main types of IUD, which are commonly used in women worldwide [13]. Beside the ones we chose, there are other IUDs available on the market, but in terms of structure and material, these are not dissimilar to the ones selected for testing in our study (Novo T is made of copper, silver, polyethylene with barium sulfate, and iron oxid (coloring of the threads); Mona Lisa is made of copper and two nylon threads; Gold Luna is made of gold, copper, and polyethylene with barium sulfate; Minerva is made of polyethylene with barium sulfate and a reservoir containing levonorgestrel and silicone, with Iron oxide used as coloring agent; the Chinese ring is made of stainless steel). For our study, the following metal containing IUDs, which form a representative sample (Table 1) were included: the “Chinese ring” IUD from China (composed of stainless steel; manufacturer company is unknown), the Gold Luna (composed of copper/gold; Dr. Schittenhelm Pharma GmbH & Co. KG, Germany), the Mona Lisa (composed of copper; Mona Lisa N.V., Heusden-Zolder, Belgium) and the Nova T (composed of copper; Bayer Pharma AG, 13353 Berlin, Germany). The Minerva IUD was not investigated, because it is made of plastic and does not contain any metal or conductive materials and is therefore not susceptible to magnetic interactions.

**Table 1:** Different types of IUD tested.

Type / Brand	Material	Dimensions (metallic coil only)	Mass of the device
Mona Lisa	Copper	diam: 2.5mm; length: 26mm	0.53 g
Gold Luna	Copper/Gold	diam: 2.5mm; length: 21mm	0.53 g
Novo T	Copper/Silver	diam: 2.5mm; length: 23.5mm	0.44 g
Chinese Ring	Stainless steel	diam: 21mm (ring); diam: 2mm (coil)	0.72 g

### Assessment of displacement force

The implants to be tested were suspended using a thin string (mass <0.015g) at the portal of the MR imaging unit (location of the strongest field gradient along the z-axis through the isocenter), and the deflection from the vertical was determined., One sample of each device was tested three times and the mean calculated. The strength of the magnetic force vector was evaluated using the following formula:

F_M=F_Gtan\alpha=m_{dev}gtan\alpha (F1)

Where m_dev_ = mass of the device, α = deflection angle, and g = gravity constant and magnetic force ${\vec{F}}_{M}$ must be orthogonal to the gravity force ${\vec{F}}_{G}$.

The attraction force is given as $\vec{F}=(\vec{m}\nabla )\vec{B}$, where $\vec{m}$ is the magnetization of the material, and $\vec{B}$, is the main magnetic field strength [14]. In case of non-ferromagnetic materials (standard IUDs are made of copper and gold) $\vec{m}$ will increase with the field strength $\vec{B}$. For implants with significant amounts of ferromagnetic materials (Chinese ring) $\vec{m}$ will change in a strong, non-linear fashion. However, at field strengths of 1.0 T and above, it is safe to assume that those materials are saturated and $\vec{m}$ will no longer increase with the B field.

Assuming the linear scaling of the magnetic force with the spatial field gradient, the magnetic force at 40T/m can be calculated as followed, taking into account that the field gradient is 4.5T/m and the field is 1.8T at the measurement location:

F_{m,40}=F_{m,4.5}\ast\frac{40\T/m}{4.5\T/m}\ast\frac{3\T}{1.8\T}\\\\ (F2)

Current 1.5 T and 3 T MR scanners have a peak field gradient on the surface of the cover of <20 T/m. Selecting twice this value allows the validity of our results for future potential increase in magnetic field strengths and magnet designs. The magnetic force *F*_*m*,4.5_ can be calculated from the deflection angle as shown by formula (F1). Because the measurement is not accurate at deflection angles close to 90°, an additional mass (plastic bottle filled with water) of 58.1g was added to the Chinese ring to reduce the deflection angle.

Out of the new magnetic force value, the expected deflection angle can be calculated as follows:

$$\alpha_{40}=tan^{-1}(tan\;\alpha_{4.5}*\frac{40T/m}{4.5T/m}*\frac{3T}{1.8T})$$ (F3)

### Torque measurements

A qualitative evaluation method of torque effects was performed by turning the IUD and each of its parts at the isocenter (in the middle of the MR tube) of the 3T magnet. The same qualitative rating (0 no torque effect to 6 strong torque effects) as mentioned in the publication by Luechinger et al. was used [14].

Torque was in addition evaluated for the Cu/Gold IUDs using the suspension Method from ASTM F2213-17. All cupper based IUDs were suspended with a low weight string (1% of IUD weight) and in the isocenter of the 3T magnet and rotated as previously described. [10]

### Assessment of heating effects

IUDs were measured in a body shaped reservoir filled with 30 liters of distilled water, 300 g of polyacrylamide (PAA) and 39.6 g of NaCl, as recently described [14, 15, 16]. The conductivity of the gel was measured as 4.7±0.4 mS/cm. The sequence for maximum specific absorption rate (SAR) as provided by the MR manufacturer was adapted by changing the number of signal averaging (NSA), the number of dynamics, and the number of slices, to achieve the minimum scan time of 15 min requested by the ASTM standard. The 3.0 T Achieva Scanner running clinical software version (Rel 3.2.3) is limited to 0.9 W/kg whole-body SAR using the RF body coil. In the research mode, the whole-body SAR limit was increased to 2 W/kg, as requested by the ASTM standard. A fiber-optic temperature setup with four MR compatible sensors from Neoptix (Quebec, Canada) was used to record the temperature changes (accuracy ± 0.1° C; temporal resolution 0.25 s). The temperature sensitive part of three sensors were placed at both ends of the conductive structures of the IUDs and placed at the boarder of the tank in the gel, while the fourth sensor was placed as a reference at the other side of the tank. The conductive structure was aligned with the main magnetic field to ensure detection of maximal heating.

During the turbo-spin echo sequences (1.5 T: FOV = 530 mm, matrix 544x512, TE = 9.8 ms, TR = 1280 ms, 2 slices, flip angle 90°, turbo factor 128, 29 NSA, 3 dynamics, scan duration 15:06min, whole-body SAR, 4.0 W/kg, B1rms = 4.53uT, forward averaged power 312±4 W; 3.0T: FOV = 530 mm, matrix 380x256, TE = 9.9 ms, TR = 2588 ms, 2 slices, flip angle 90°, turbo factor 128, 30 NSA, 3 dynamics, scan duration 15:47 min, whole-body SAR, 2.0 W/kg, B1rms = 2.44 uT, forward average power 129±2 W) the temperature changes were continuously measured. The metallic coils have a length (in case of the ring, a diameter) of up to 26mm. The coil of the copper IUDs is made out of up to 60 windings resulting in an estimated wire length of about 20cm, which could be a critical length at 1.5T and 3T. However, the wire is not isolated and the windings touch each other’s and therefore the coil length is the more relevant length for RF heating. A coil length of <3cm can be considered electrically short for all field strength up to 3T.

### Measurement of artifacts

**Fig 1:**
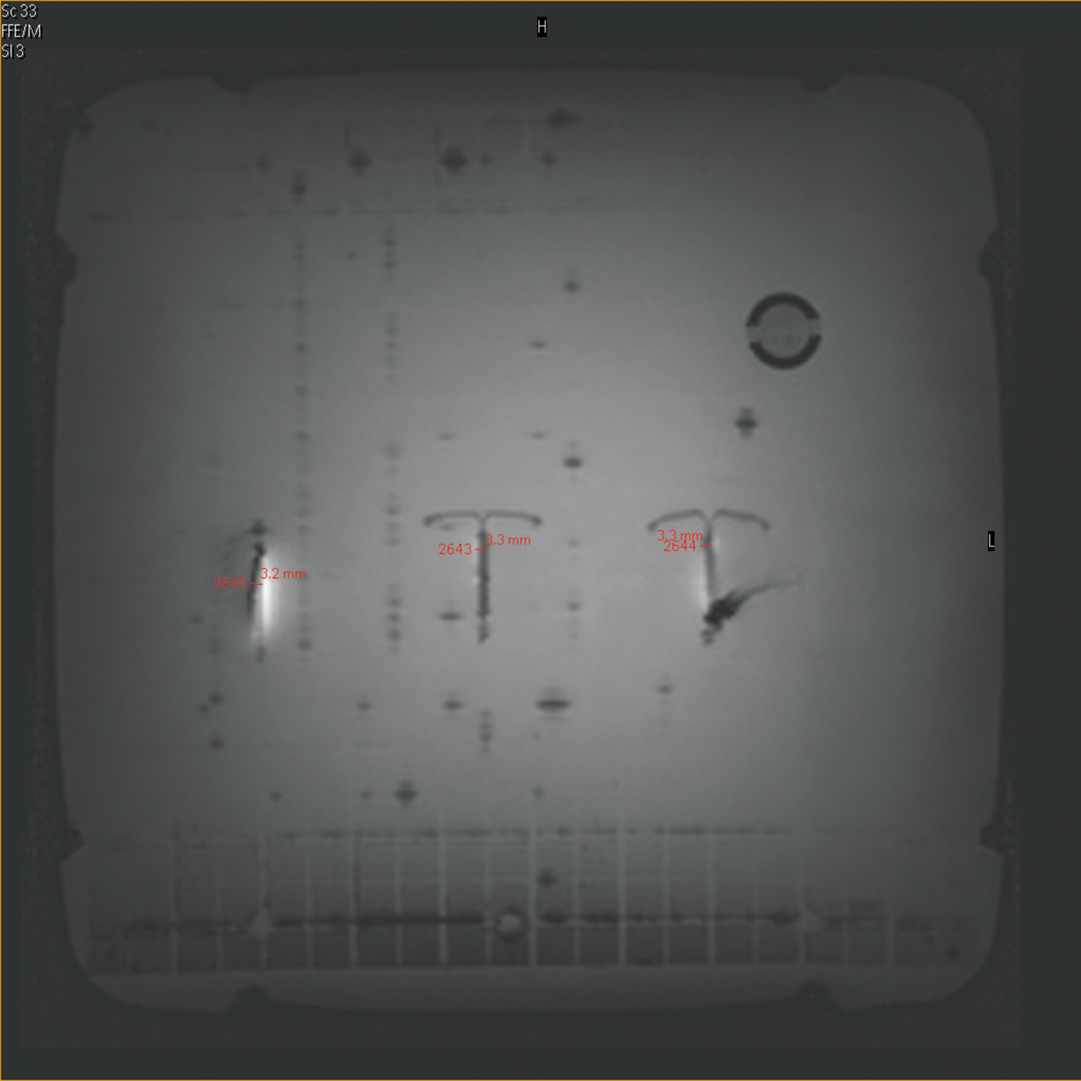
The gold/copper IUD in the filled tank: The artifacts of the three copper/gold IUD when imaged with the spin-echo sequence at 3.0 T.

Assessment of artifacts caused by the implants were performed on the 3.0 T Philips system. The IUDs were placed in a tank filled with ~8 liters of copper sulfate solution (1 g CuSO_4_ per liter distilled water; 0.1%). Two orientations of the implants relative to the main magnetic field were tested. For the orthogonal test, the tank was rotated 90° counterclockwise. The parts were placed on a supportive grid in the center of the tank (Fig 1). The grid is made out of an acrylic glass block, with alternating 1 mm and 2 mm large cuts. The cubes between two cuts have 1 cm side length. The acrylic glass grid was used as reference object as specified by the ASTM standard. The size of the artifacts were measured using a gradient-echo (FOV = 256 mm, matrix 256x256, slice thickness 3 mm, TE = 15 ms, TR = 100 ms, 4–8 slices, flip angle 30°, Band width 32.4 kHz) and spin-echo (FOV = 256 mm, matrix 256x256, slice thickness 3 mm, TE = 20 ms, TR = 500 ms, 4–8 slices, flip angle 90°, Band width 32.4 kHz) sequence. The slice orientation and the fold-over direction of the imaging sequences were altered to differentiate all effects.

## Results

### Displacement force measurements

**Fig 2:**
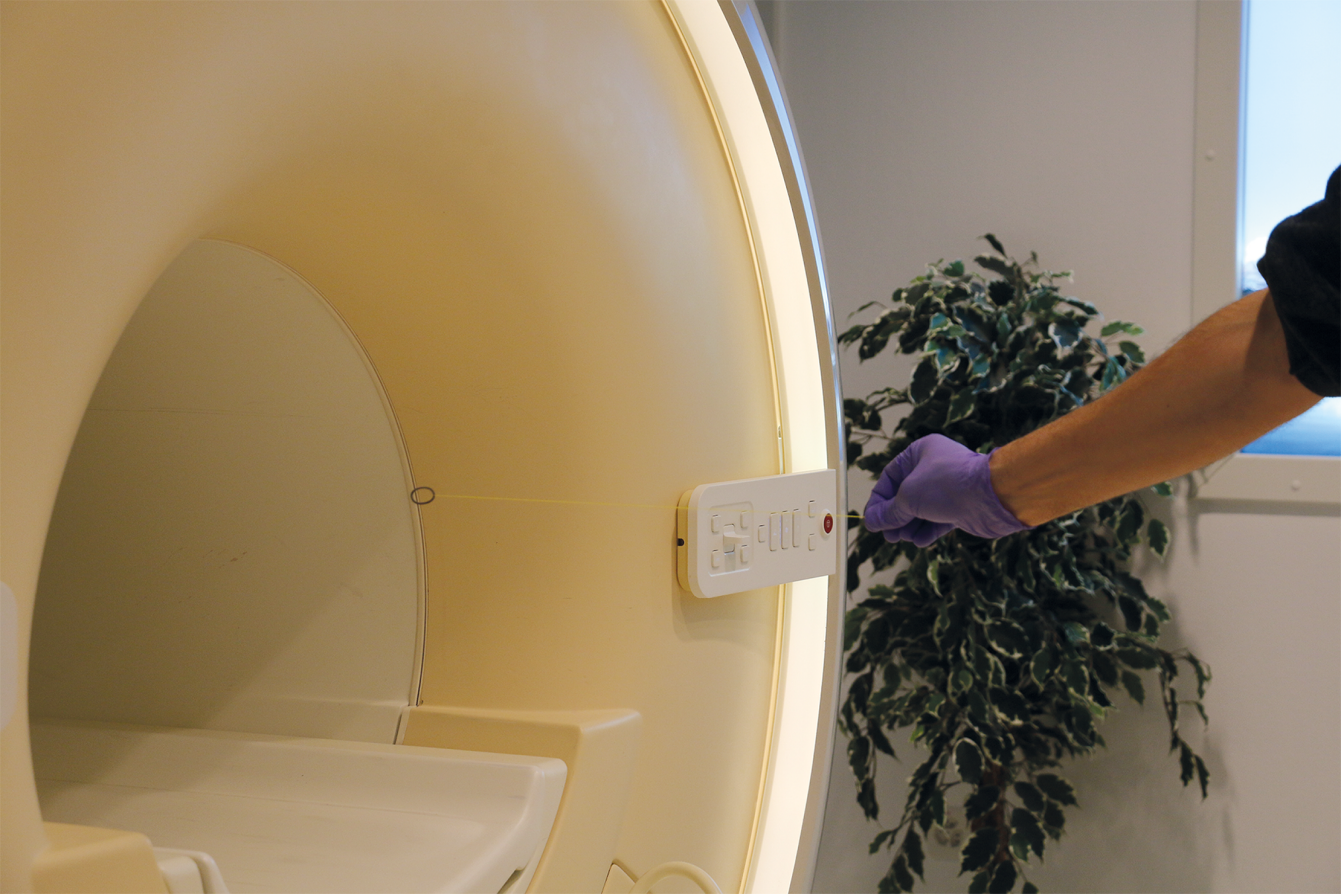
The stainless steel “Chinese ring”: The “Chinese Ring” held close to the MRI (3.0 T) demonstrating the high induced magnetic force.

The deflection angles of the three copper/gold IUD were close to zero (0.5° ± 0.5° of deflection for Mona Lisa, Gold Luna and Nova T). Extrapolated to 40 T/m (to keep a safety margin for future developments) the deflection angle will correspond to 7° ± 7° and the magnetic force will be < 1 mN at the worst-case location (Table 2). By comparison, the gravitational force exerted on the three copper IUD is > 4 mN. The deflection angle of the Chinese ring (mass 0.72 g) after addition of an extra weight of 58.1 g was 41° ± 1° in the main magnetic field gradient of 4.5T/m, which results in a magnetic force of 7.6 N on the Chinese ring. Due to the high force, no extrapolation to a stronger field gradient was performed. (Fig 2).

**Table 2:** IUD safety measurements on 1.5 T and 3.0 T: Overview of displacement force, torque effects (0: no torque, 6: highest torque) and diameter of imaging artifacts.

	displacement force	Torque effects	artifact diameter	
IUD	3.0 T and 40T/m	3.0 T	gradient echo	spin echo
	[mN]	[0–6]	3.0 T [mm]	3.0 T [mm]
Mona Lisa	0.5	0	3.8	3.8
Gold Luna	0.5	0	3.8	3.8
Novo T	0.5	0	3.8	3.8
Chinese Ring	7600 (4.5T/m)	6	200	150

### Torque effect measurements

Manual rotation: The standardized manual rotation of the IUDs at the isocenter of the 3.0 T magnet, showed no torque effect for the three copper IUDs, while a strong torque effect was observed for the Chinese ring (scored as 6). In clinical practice, a rotation effect within the patient is very likely for the Chinese ring, and tissue damage is a potential risk.

Suspension Method: no movement or alignment to the static magnetic field was observed for any of the cupper/gold IUD. The test was not performed for the stainless steal ring, due to the known high torque effects.

### Measurements of heating effects

The measurements in the 1.5 T MRI system (measured with a SAR of 4 W/kg) yielded a maximum temperature increase (no subtraction of background heating was applied) of 1.4° C for the Chinese ring, of 3.2° C for the Gold Luna, of 3.3° C for the Mona Lisa, of 3.8° C for the Nova T and of 2.2° C for the filled reservoir without an IUD, following measurements for 15 minutes, respectively. For the 3.0 T MRI measurements (measured with a SAR of 2 W/kg) the maximum temperature increase was doubled and thus normalized to a SAR of 4 W/kg body-weight. The recalculation (SAR of 4 W/kg) yielded temperature increases of 3.4° C for the Chinese ring, 3.8° C for the Gold Luna, 4.8° C for the Mona Lisa, 4.8° C for the Nova T and 3.6°C for the filled tank without an IUD for a scnning time of 15 minutes. All results are summarized in Table 3.

**Table 3:** Results of the heating effects with maximum temperature increase over 15 min.

IUD	Temperature increase [°C]		Temperature increase [°C]	
	1.5 T; SAR 2 W/kg**	1.5 T; SAR 4 W/kg*	3 T; SAR 2 W/kg*	3 T; SAR 4 W/kg**
Reference	1.1	2.2	1.8	3.6
Gold Luna	1.6	3.2	1.9	3.8
Mona Lisa	1.7	3.4	2.4	4.8
Nova T	1.9	3.8	2.4	4.8
Chinese ring	0.7	1.4	1.7	3.4

*measured values

**calculated values

### Artifact size

**Fig 3:**
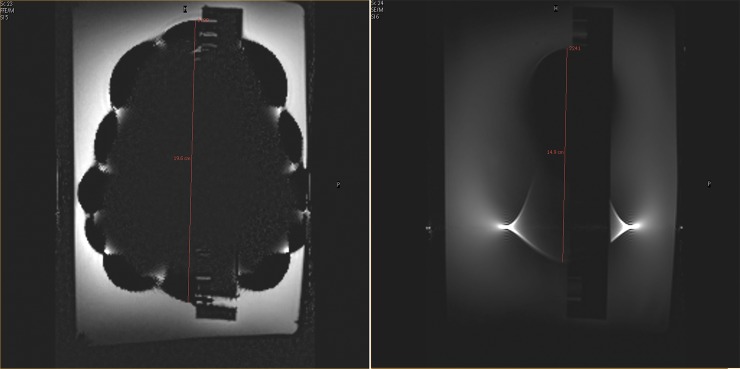
Artifact of the ferromagnetic stainless steel IUD (Chinese ring): Artifact of the “Chinese ring” on a gradient echo sequence (3.0 T) on the left, exemplified by signal void and image distortion. Note water surface (left side) and bottom of the tank (right side). Artifact induced by the stainless steel IUD (Chinese ring) on spin echo sequence (3.0 T) is as shown on the right.

The three copper IUD (NovaT, Mona Lisa and Gold Luna) in the 3.0 T MRI resulted in an artifact diameter of 4 mm ± 1 mm. (Fig 1). Taking the 3 mm diameter of the copper coil into account, these artifacts should not propagate for more than 1 mm beyond the surface of the implant. However, the artifact induced by the stainless steel IUD (Chinese ring) leads to an artifact diameter of 200 mm ± 10 mm using the gradient echo pulse sequence setting and to an artifact diameter of 150 mm ± 10 mm using the spin-echo sequence (Fig 3).

## Discussion

Our results show that the three frequently used IUDs (Mona Lisa containing copper; Gold Luna containing copper/gold and Novo T containing copper/silver) can be regarded as MR conditional (no known hazards in a specified MR environment) for their safety at 1.5T and 3.0T, even when considering a whole body SAR of up to 4W/kg and a static magnetic field gradient of up to 40T/m. Our results indicate that the main magnetic field gradient does not induce significant movement related to the magnetic force compared with the gravitational force in these implants. In addition, no significant heating was observed although the RF hearting effects at <3.0T were not tested. Due to the small conductive structure (metallic coil) of these IUDs, it is likely that only limited RF heating will occur at the lower field strengths (< = 1T).

By contrast, the so called Chinese IUD ring composed of stainless steel not only generates pronounced artifacts, but also experiences significant dislocation in the applied magnetic fields (deflection angle without additional weight: ~90°). Since the induced force and torque effects pose a real safety risk, the device should be considered as MR unsafe.

The issue of IUD’s translocation following MRI examinations is worth considering, as this may require clinical follow-up to control or re-position the IUD after MRI [8].

In view of our results, copper/gold/silver containing IUDs appear to be clinically safe with MRI effects being significantly less than earth’s gravitational force, in contrast to the stainless steel containing IUD (Chinese IUD ring), which are MR unsafe. To our knowledge there are no prior publications evaluating the safety of the Chinese ring IUD. This IUD is susceptible to MR induced motion, and hence undesired movement within the uterus or displacement into the vagina. As a result, patients carrying the Chinese ring IUD should be considered as contraindicated for MRI examination of any body region. In case of a strong indication for MRI in these women, removal of the IUD prior to MRI would be strongly recommended because of the potential for the device to degrade images, as well as the high risk for displacement.

Our results also confirm the findings of previous IUD compatibility studies and IUD device listings [17], stating that most IUDs are MR safe or conditional. The stainless steel Chinese IUD rings are however not mentioned within this database, even though this is one of the most frequently used contraceptive devices in China.

Due to the increased mobility of people as a result of tourism and migration, patients with the Chinese ring IUD implant may require MR examination in other parts of the world. Hence, the potential harm to these patients undergoing any MRI studies should be considered. To our knowledge, even though the manufacturing of the stainless steel Chinese IUD ring has been stopped around the year 2000, the longevity of these devices means that the implant is left in situ in a significant number of Chinese patients. Furthermore, there are indication that ferromagnetic IUDs in other forms may still be available on the market. It also needs to be emphasized that Chinese women have the highest prevalence of employing birth control measures (85% in comparison to 79% in the USA [5]). Moreover, IUDs are the most frequently used form of contraception in about 40.6% of all women compared with approximately 2.1% of women in the USA [4]. This socio-geographical difference may be accounted by the relatively low price of the IUDs in China, the social one-child policy, high Pearl-Index (0.1–1.5) and high adherence [4,18].

To our knowledge, this is also the first study to systematically investigate according to international ASTM standards the properties of frequently used IUDs at 1.5 T and 3.0 T MRI with respect to heating, magnetic force, torque effects and induced artifacts. Previous studies only investigated the heating effects of IUDs at 1.5 T MRI, which corroborates well with our results [19]. Due to the about 10 fold smaller magnetic acceleration forces compared with the gravitational force in a worst case field gradient of 40 T/m, the authors do not see any justification to check the IUD placement of copper/gold IUD of such patients following an MRI examination.

Our study has the following limitations. Firstly, only a limited number of IUDs were included as it was not possible to retrieve specific devices limited to certain countries or regions. However, we included the most representative IUDs composed of non-ferromagnetic copper/gold/silver that were available to us, as well as the most frequently used IUD type used in China. Due to similar metal winding techniques used in other IUDs, we expect other similar devices to behave in a comparable manner. Secondly, we could not obtain the full technical information of the Chinese stainless steel IUD due to language barrier and lack of manufacturer information. Thus, the full metal composition of the device besides stainless steel could not be verified. Lastly, our present study is an in-vitro assessment, by which the findings are being extrapolated to the in-vivo scenario. However, the effects due to secondary changes after device placement could also not be assessed in the present study. Additional MR safety issues such as the induction of eddy currents or nerve stimulation were also not assessed.

For relevant medical devices, the MR safety evaluations should be performed by the implant manufacturers in order to determine in a liable form patient safety for MR scanning. Even so, the tests performed in this study follow the same test standards as requested for certification labeling by the manufacturer. However, our tests can not replace those conducted by the device manufacturers. It would be preferable that all medical implants would contain an official MR safety label in their instruction for use, as this would allow MR technicians and radiologists to have greater confidence in the imaging of these patients.

## Conclusions

The tested cupper/gold/silver IUDs can be considered as MR conditional at 1.5T and 3.0 T, up to a wbSAR of 4W/kg, and a main magnetic field gradient of up to 40 T/m, which results in only about 1 mm imaging artifacts from the implant surface. However, the ferromagnetic Chinese stainless steel IUD, one of the most frequent IUD used in China, not only strongly hampers pelvic MR imaging due to extensive induction of artefacts, but is potentially harmful to patients due to high magnetic dislocation forces. Therefore, stainless steel IUD should be regarded as MR unsafe. Patients with such devices in-vivo should be regarded as contraindicated for any MRI examination.

## Supporting information

S1 File (ZIP)
