# Supplementary material for: Safety of intrauterine devices in MRI
Source: PLoS One. 2018 Oct 9;13(10):e0204220. doi: 10.1371/journal.pone.0204220 (PMC6177157; doi:10.1371/journal.pone.0204220)
Supplement: S1 File — (ZIP) [file pone.0204220.s001.zip › artifacts/summary_artifacts.pdf]

Implant (conductive structure) parallel to main magnetic field.

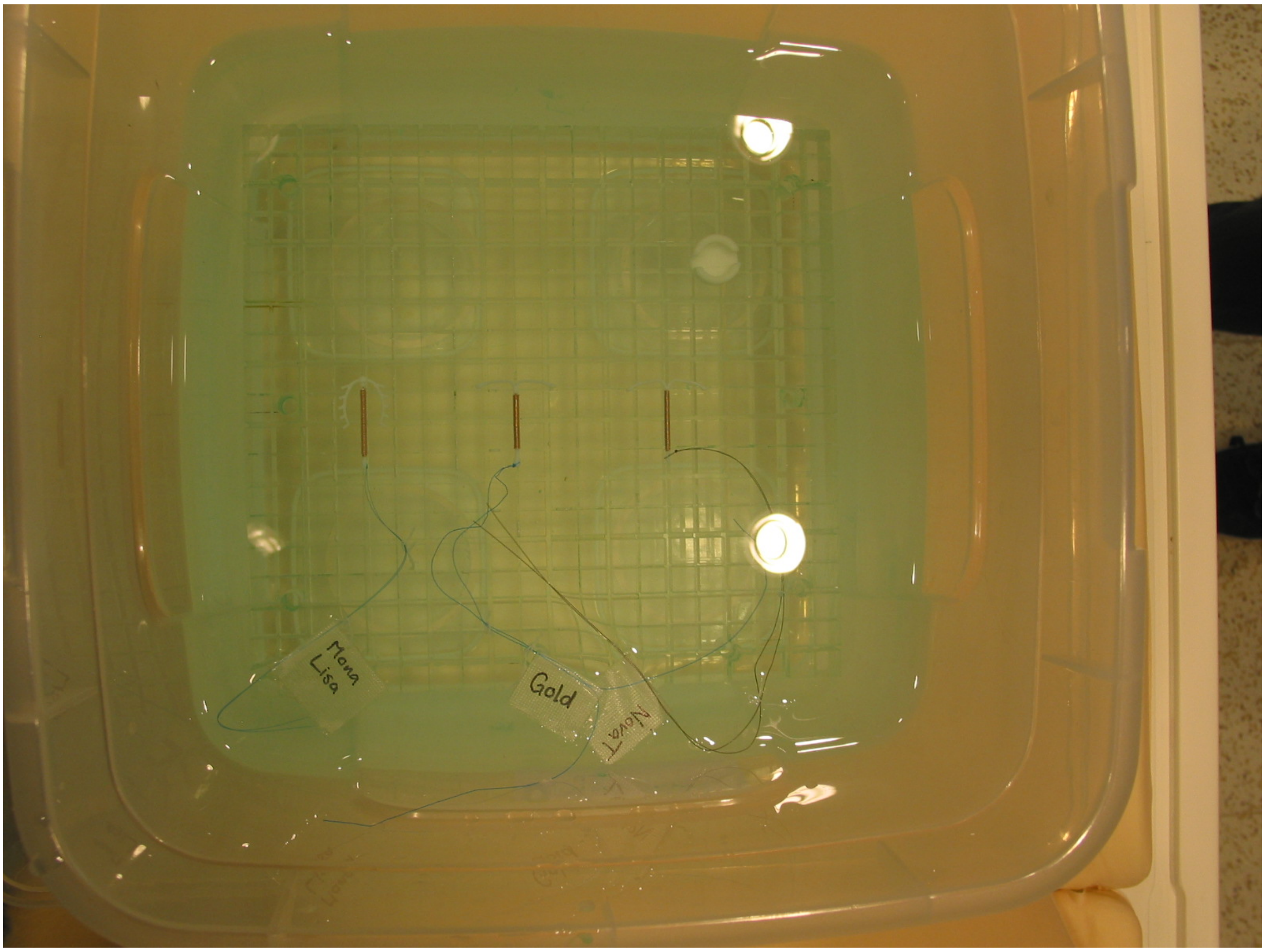

From left to right: Mona Lisa, Gold and Nova T.

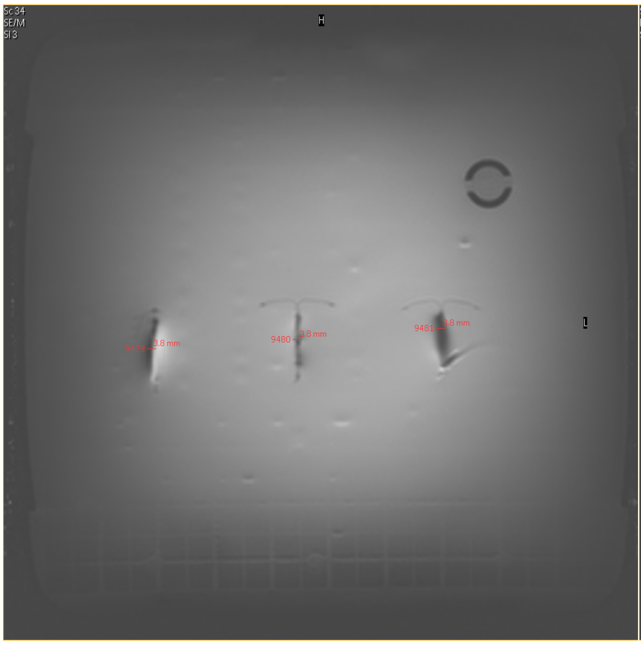

Gradient echo Coronal RL (Phase enc.)

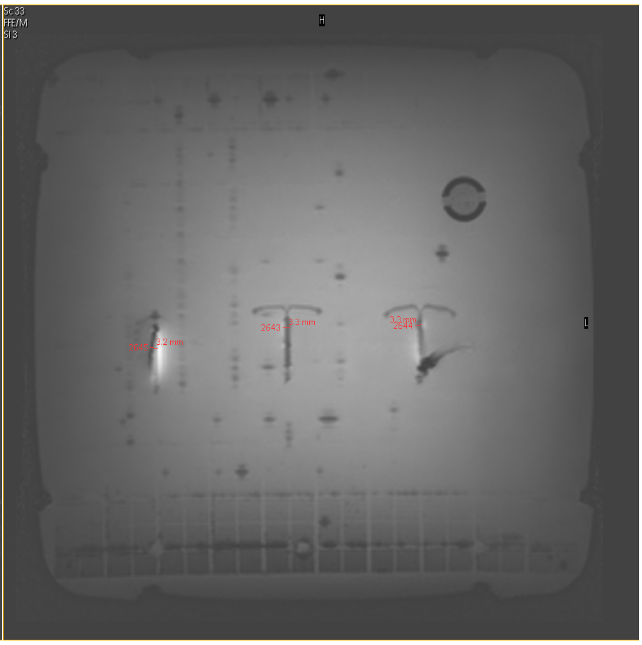

Spin echo Coronal RL (Phase enc.)

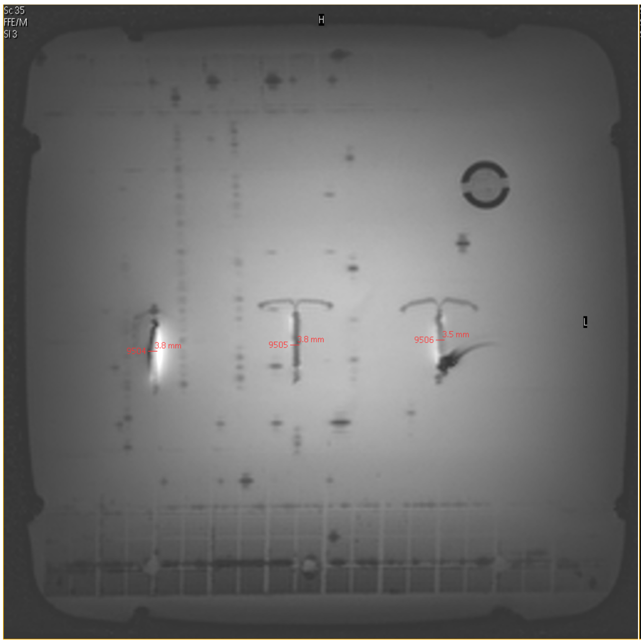

Gradient echo Coronal FH (Phase enc.)

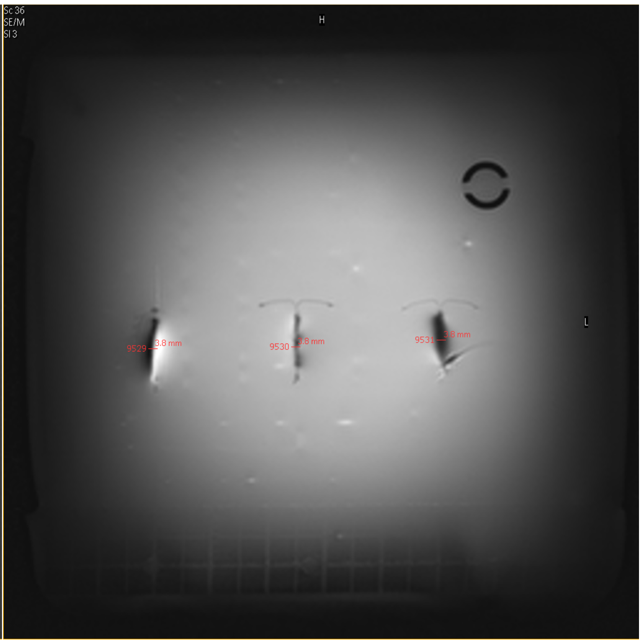

Spin echo Coronal FH (Phase enc.)

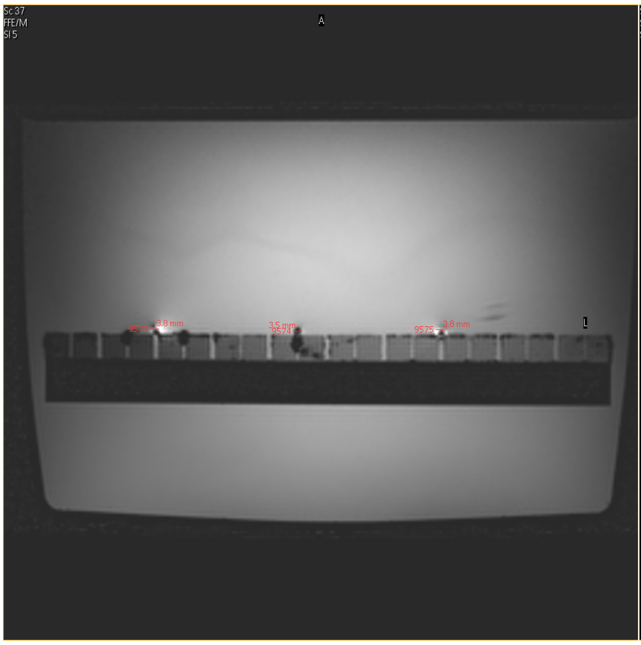

Gradient echo Axial AP (Phase enc.)

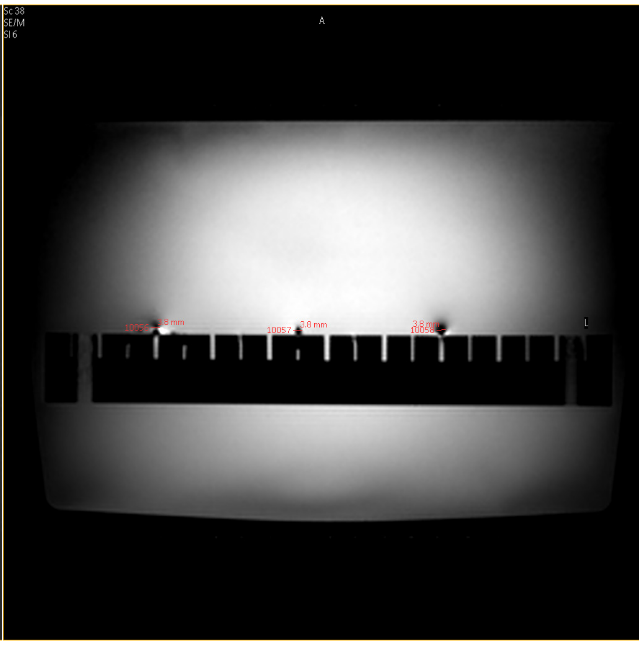

Spin echo Axial AP (Phase enc.)

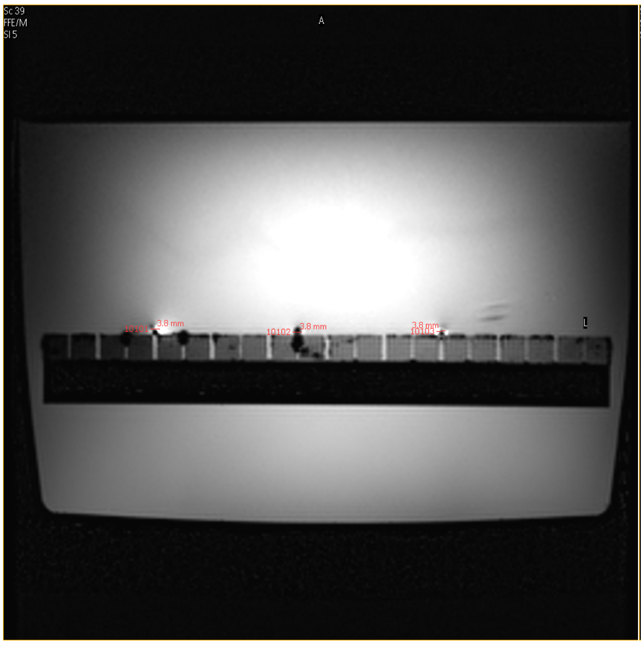

Gradient echo Axial RL (Phase enc.)

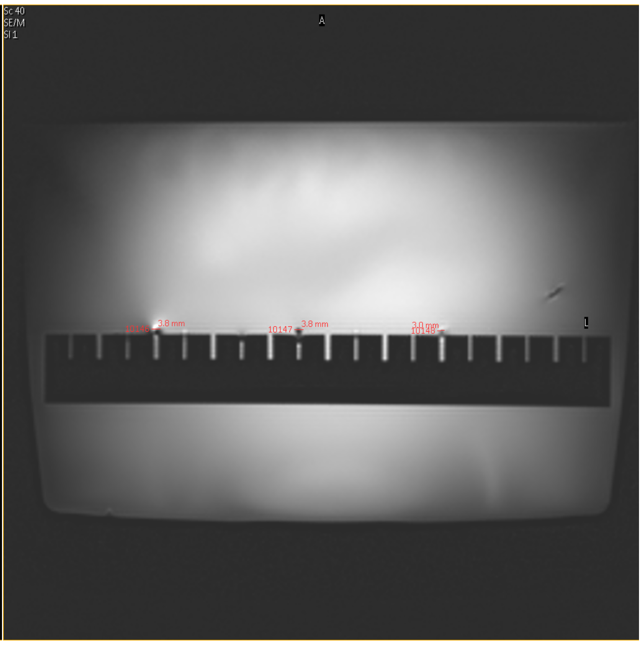

Spin echo Axial RL (Phase enc.)

Since the conductive spiral is aligned with the B0 field and therefore with the E-field, higher B1 fields will flow on the conductive structure and results in additional B1 artifacts (which are not taken into account for the susceptibility artifacts). To avoid the B1 artifacts, the spirals have been rearranged to reduce those artifacts.

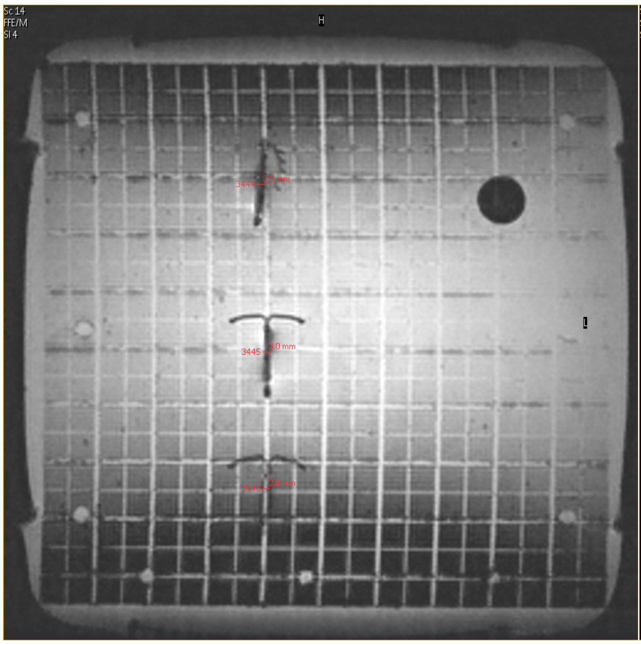

Gradient echo Coronal FH (Phase enc.)

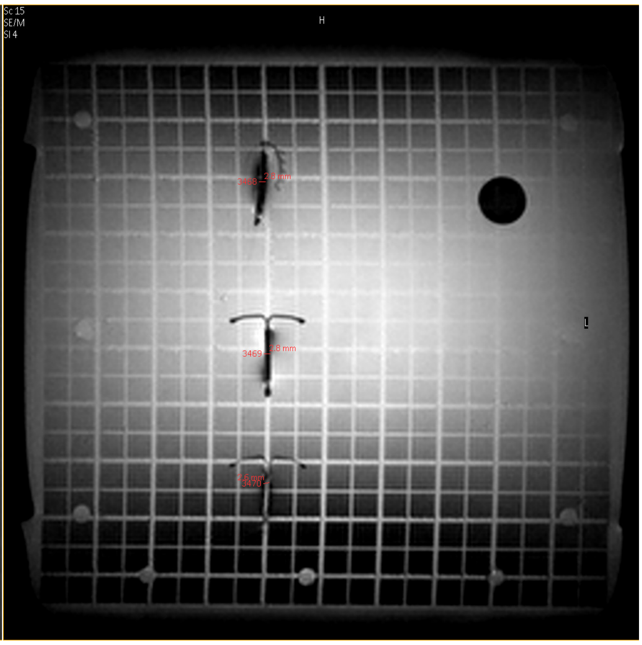

Spin echo Coronal FH (Phase enc.)

Implant orthogonal to main magnetic field

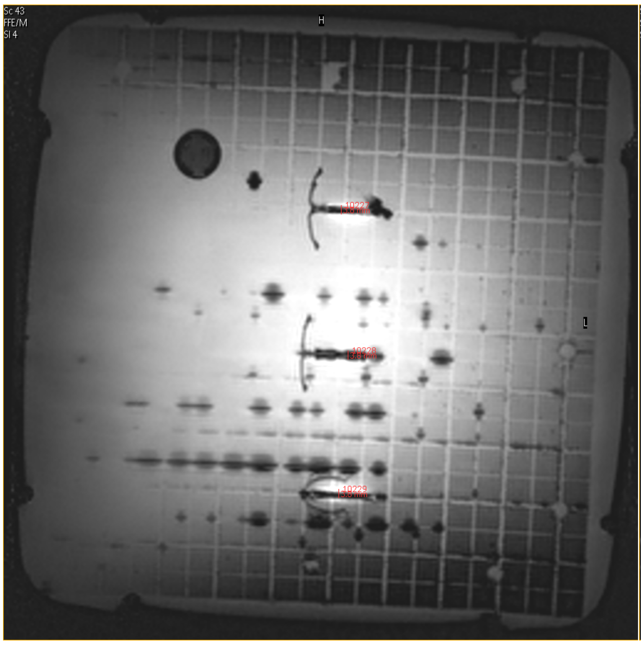

Gradient echo Coronal RL (Phase enc.)

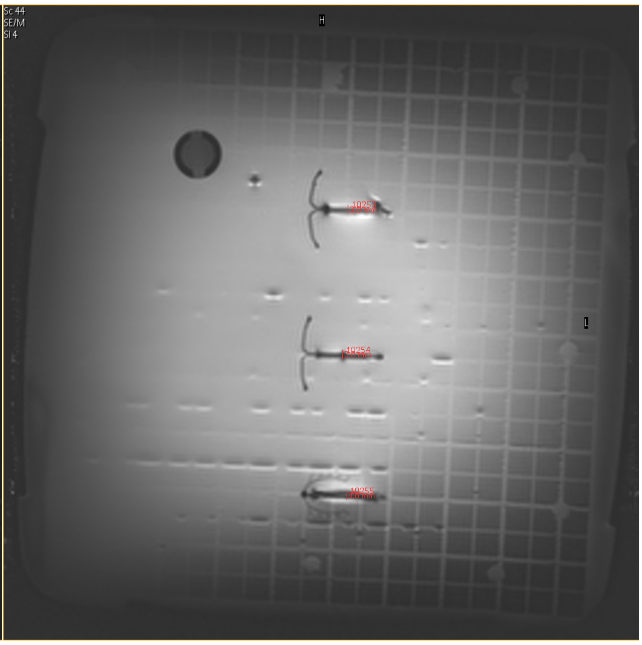

Spin echo Coronal RL (Phase enc.)

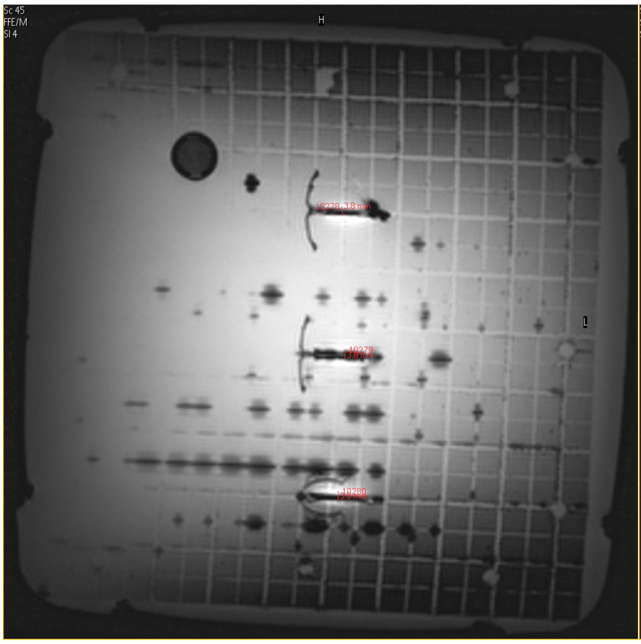

Gradient echo Coronal FH (Phase enc.)

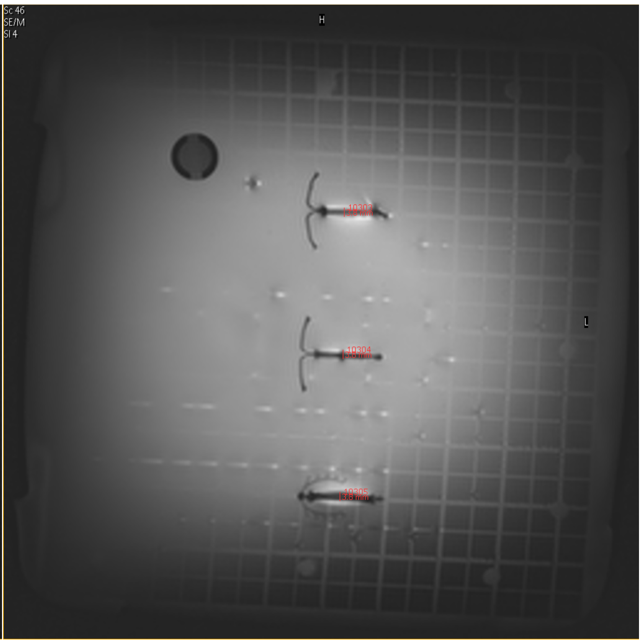

Spin echo Coronal FH (Phase enc.)

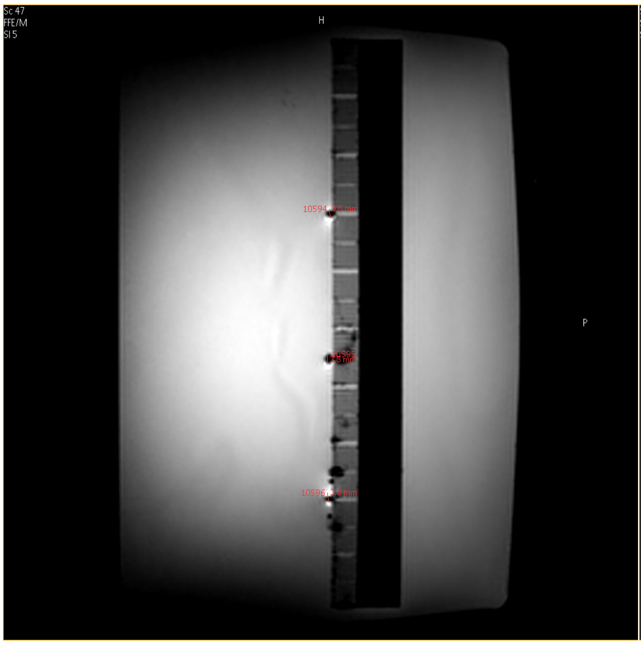

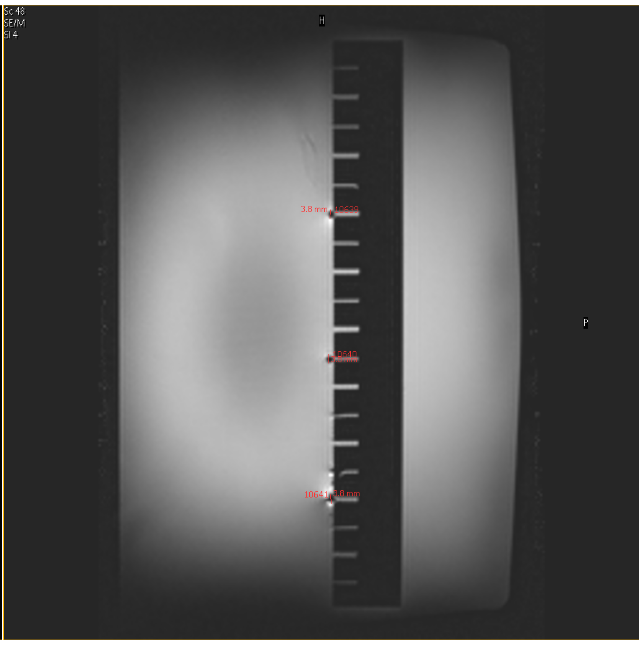

Gradient echo Sagittal AP (Phase enc.)

Spin echo Sagittal AP (Phase enc.)

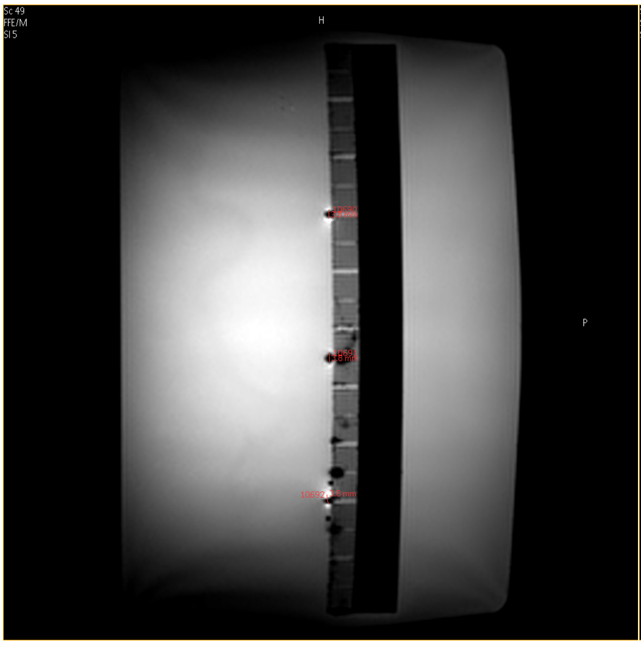

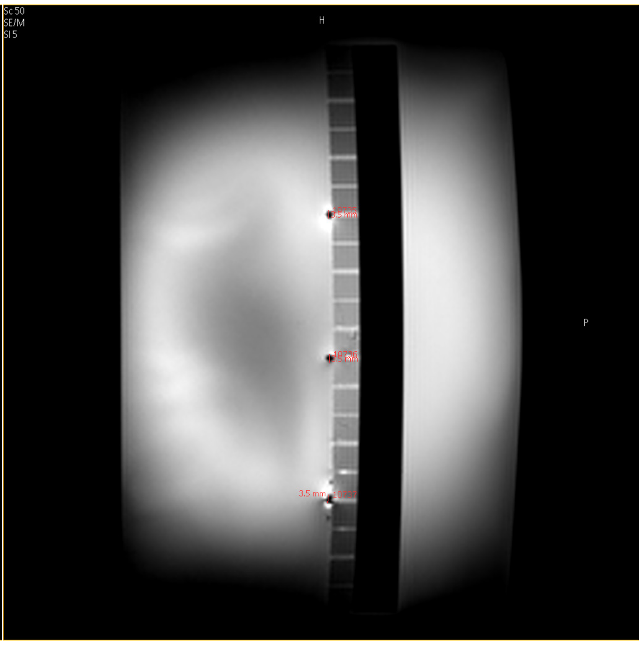

Gradient echo Sagittal FH (Phase enc.)

Spin echo Sagittal FH (Phase enc.)

Chinease Ring:

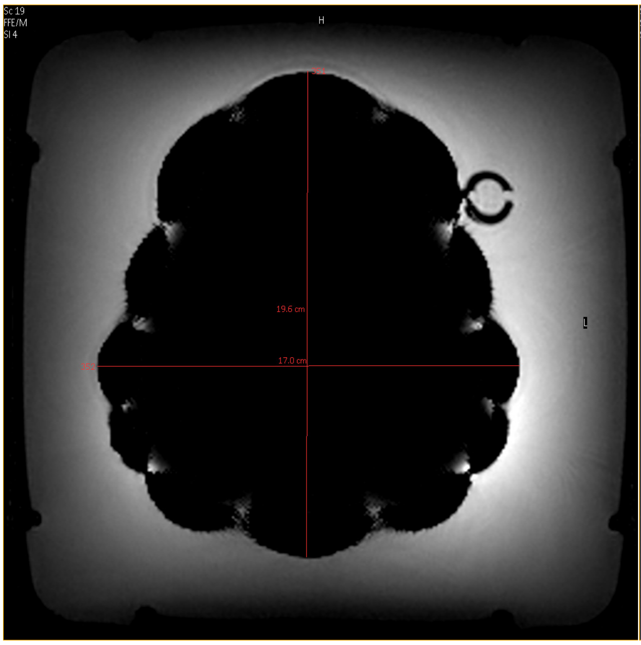

Gradient echo Coronal RL (Phase enc.)

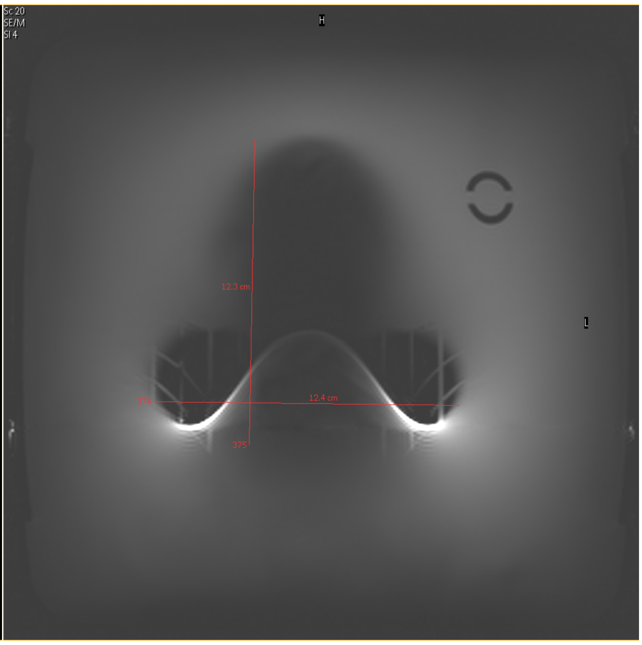

Spin echo Coronal RL (Phase enc.)

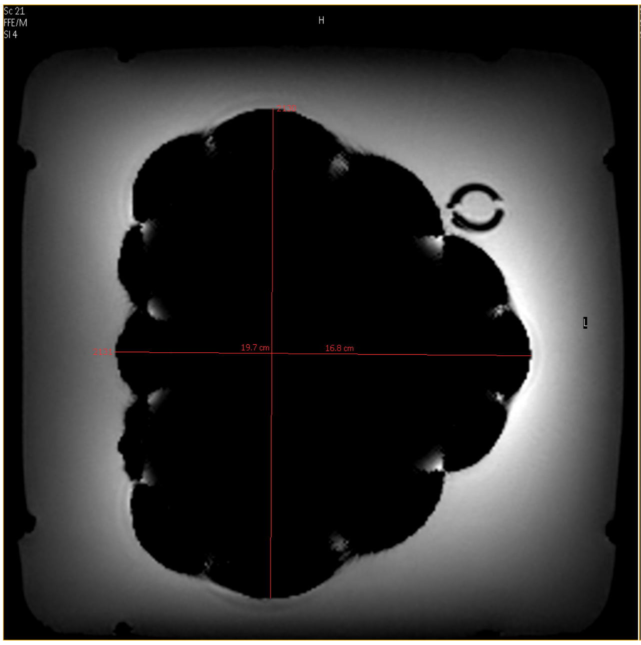

Gradient echo Coronal FH (Phase enc.)

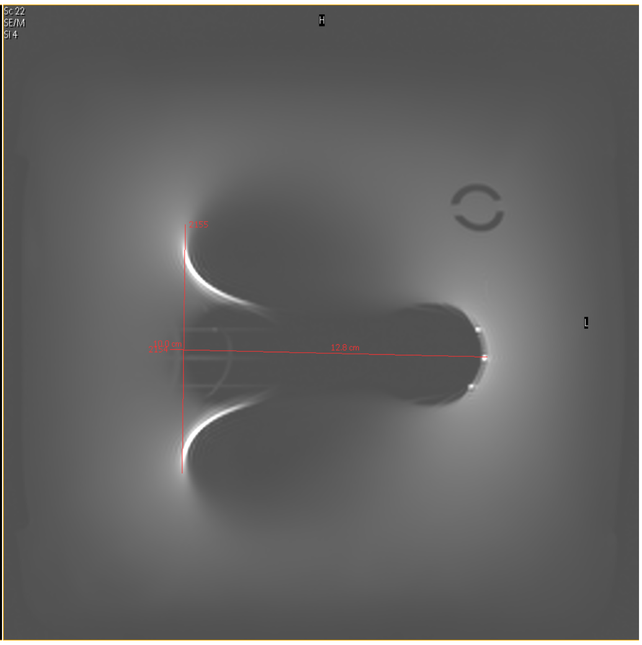

Spin echo Coronal FL (Phase enc.)

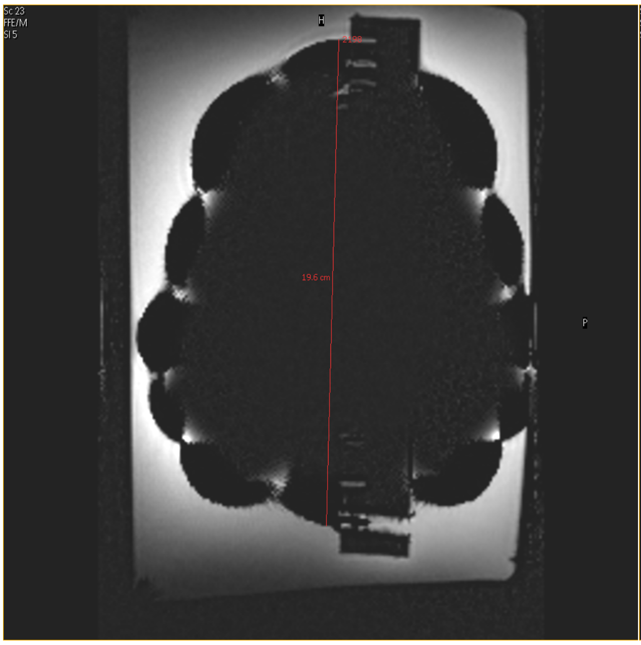

Gradient echo Sagital AP (Phase enc.)

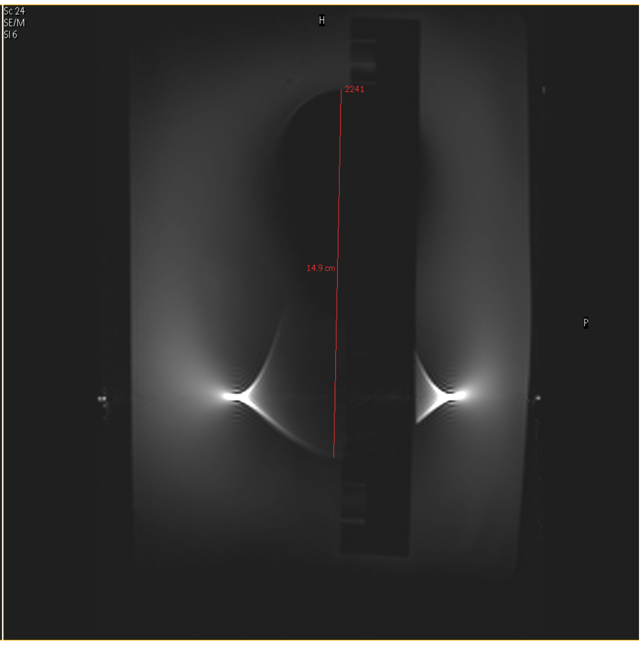

Spin echo Sagital AP (Phase enc.)

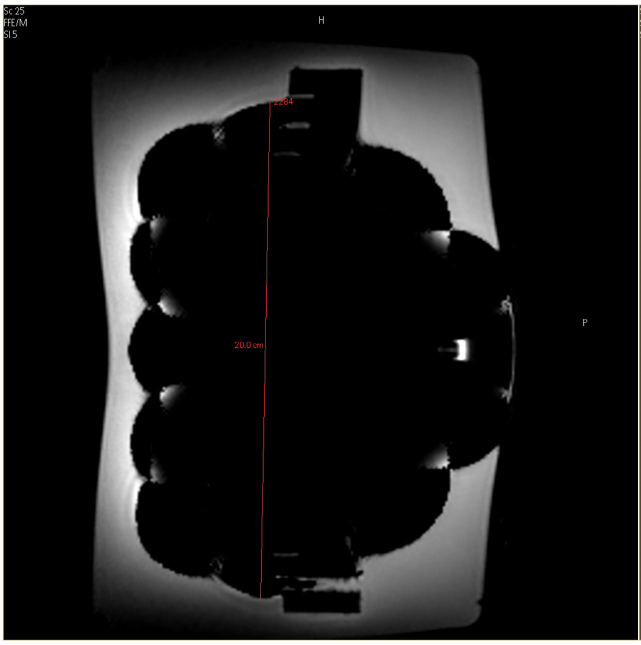

Gradient echo Sagital FH (Phase enc.)

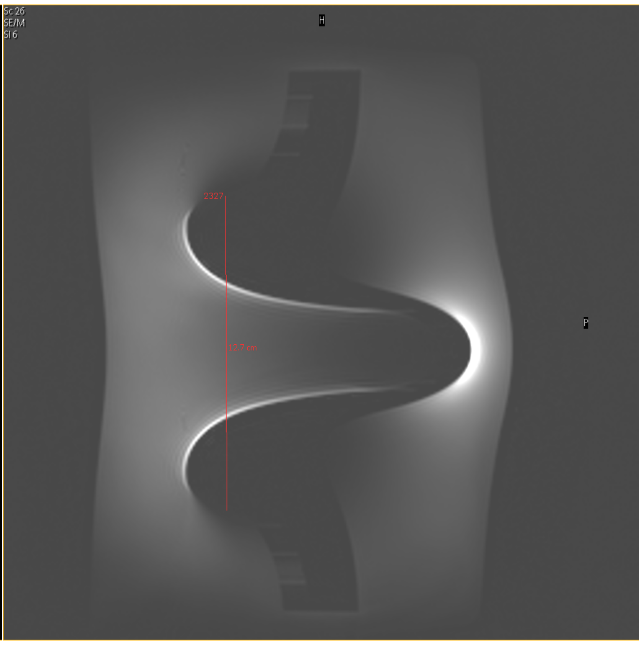

Spin echo Sagital FH (Phase enc.)

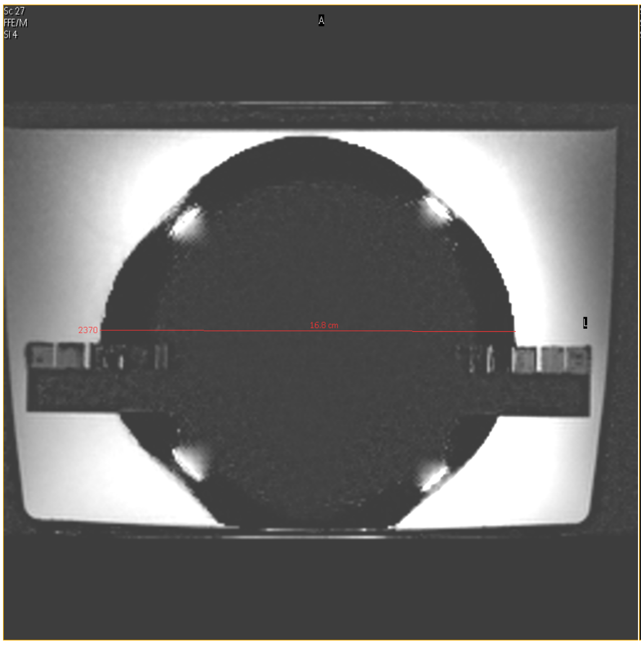

Gradient echo Transversal AP (Phase enc.)

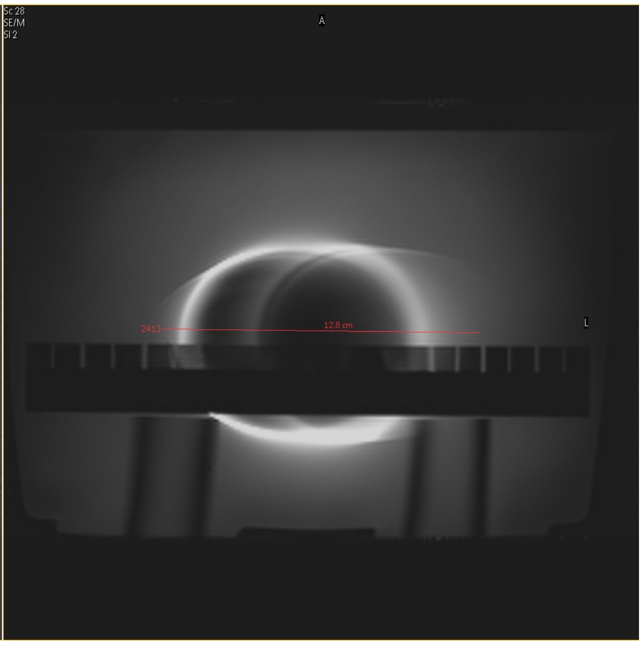

Spin echo Transversal AP (Phase enc.)

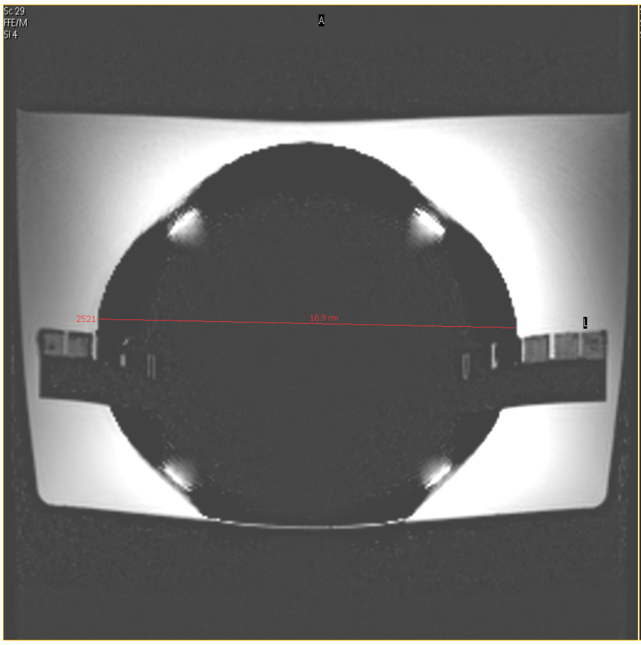

Gradient echo Transversal RL (Phase enc.)

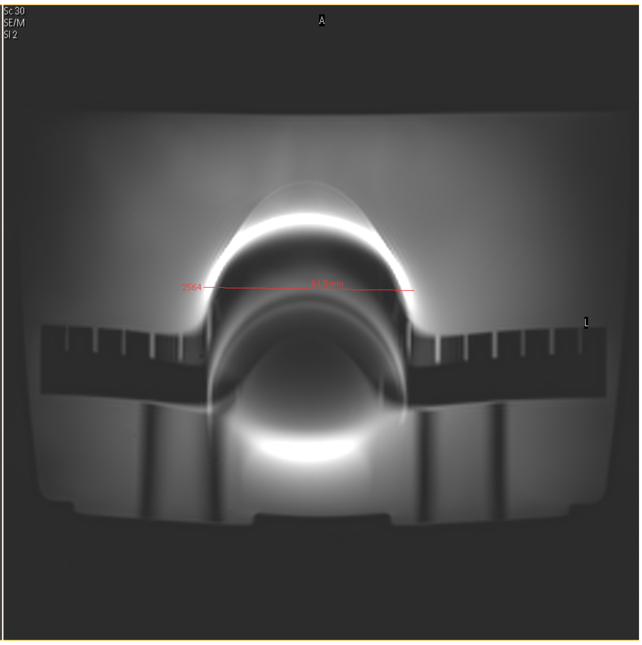

Spin echo Trasnversal RL (Phase enc.)
