# Supplementary figures and images for: Safety of intrauterine devices in MRI
Source: PLoS One. 2018 Oct 9;13(10):e0204220. doi: 10.1371/journal.pone.0204220 (PMC6177157; doi:10.1371/journal.pone.0204220)

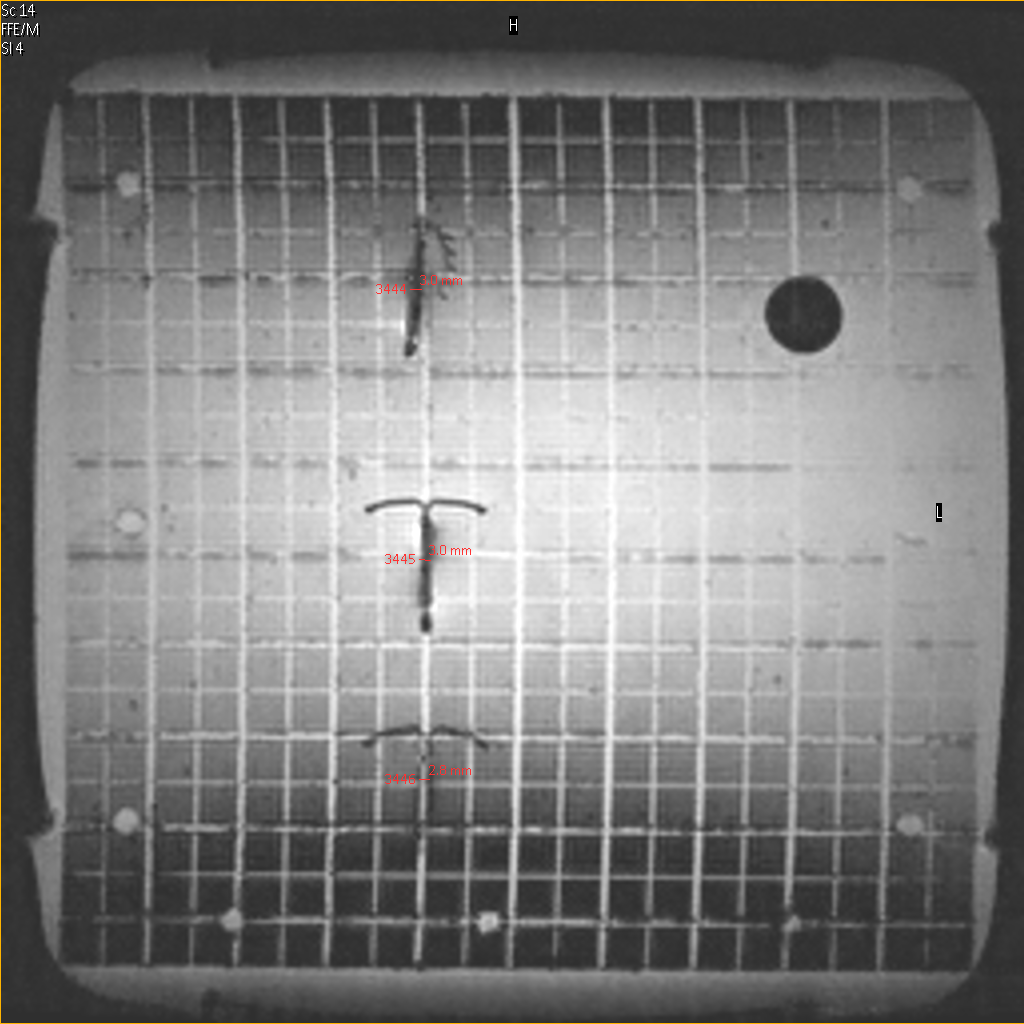

Supplement: S1 File — (ZIP) [file pone.0204220.s001.zip › artifacts/S1.png]

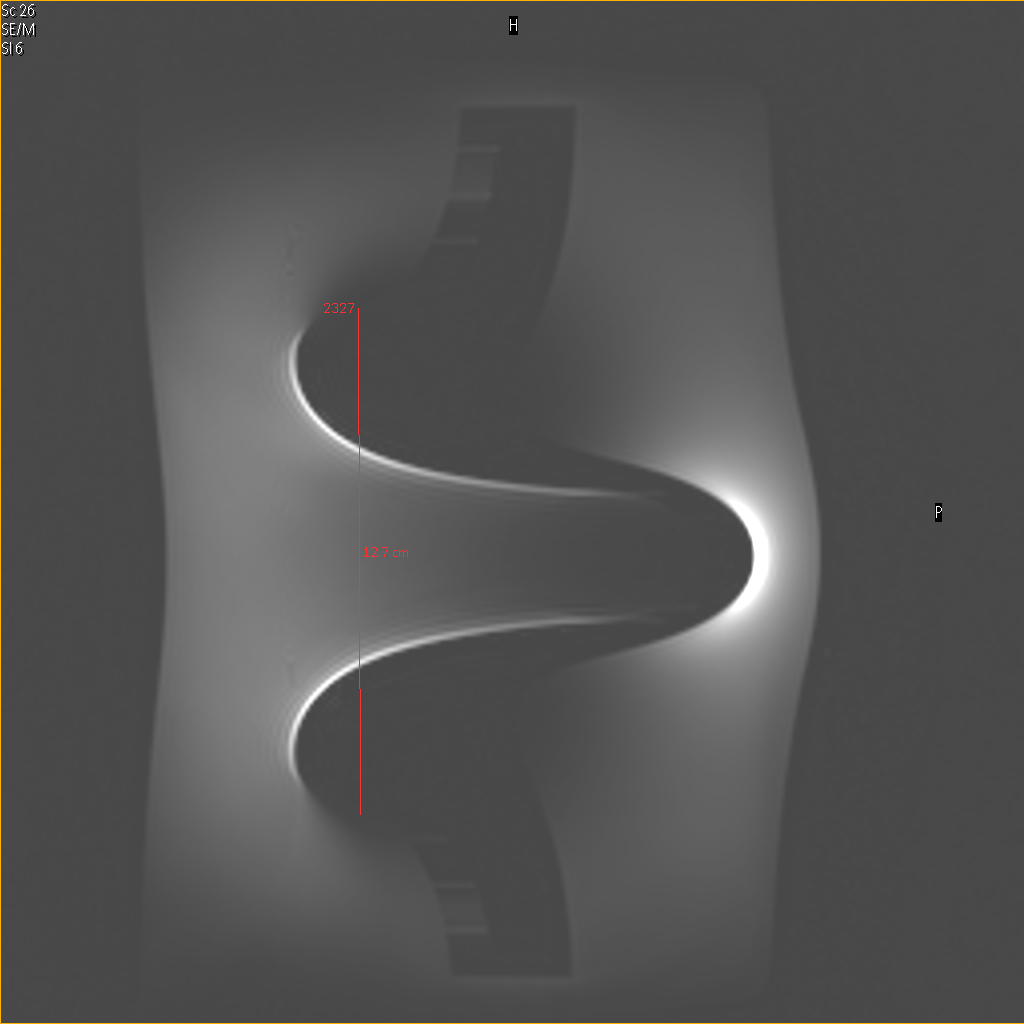

Supplement: S1 File — (ZIP) [file pone.0204220.s001.zip › artifacts/S10.png]

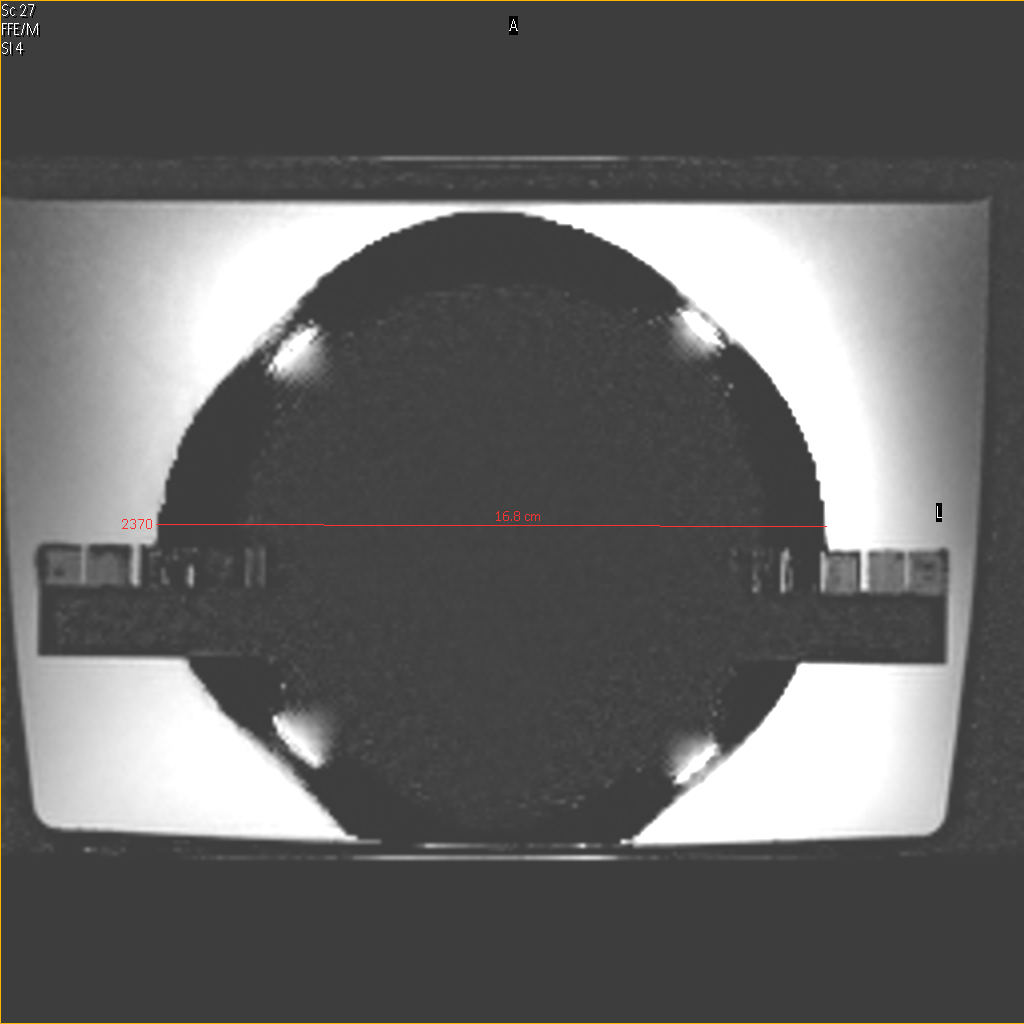

Supplement: S1 File — (ZIP) [file pone.0204220.s001.zip › artifacts/S11.png]

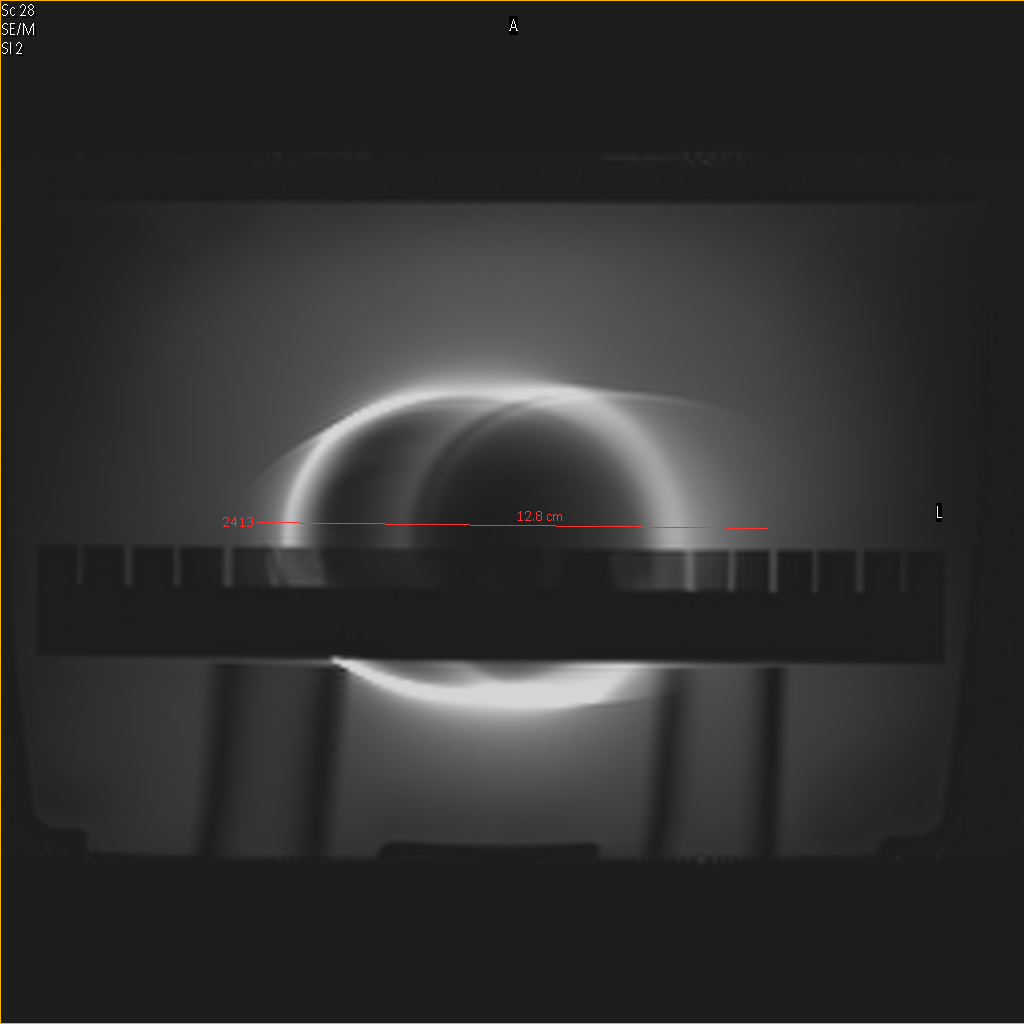

Supplement: S1 File — (ZIP) [file pone.0204220.s001.zip › artifacts/S12.png]

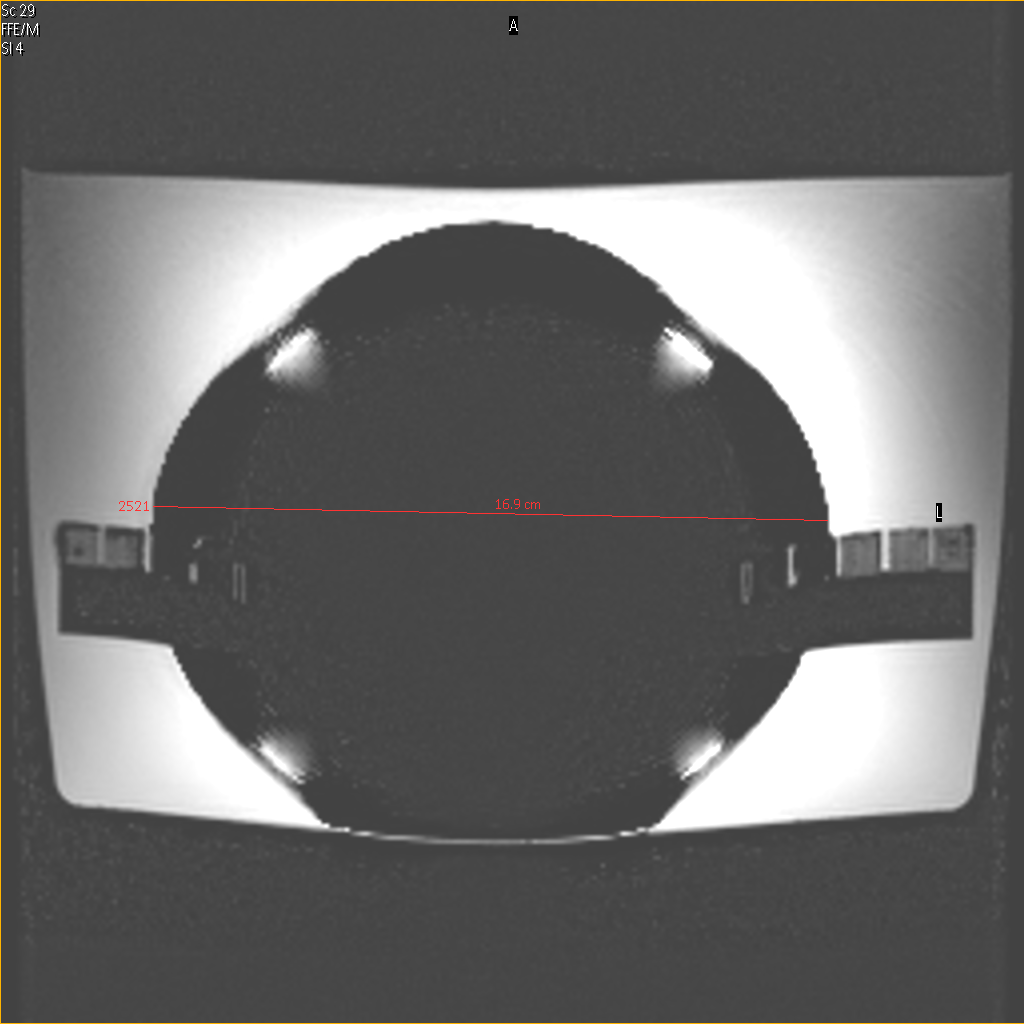

Supplement: S1 File — (ZIP) [file pone.0204220.s001.zip › artifacts/S13.png]

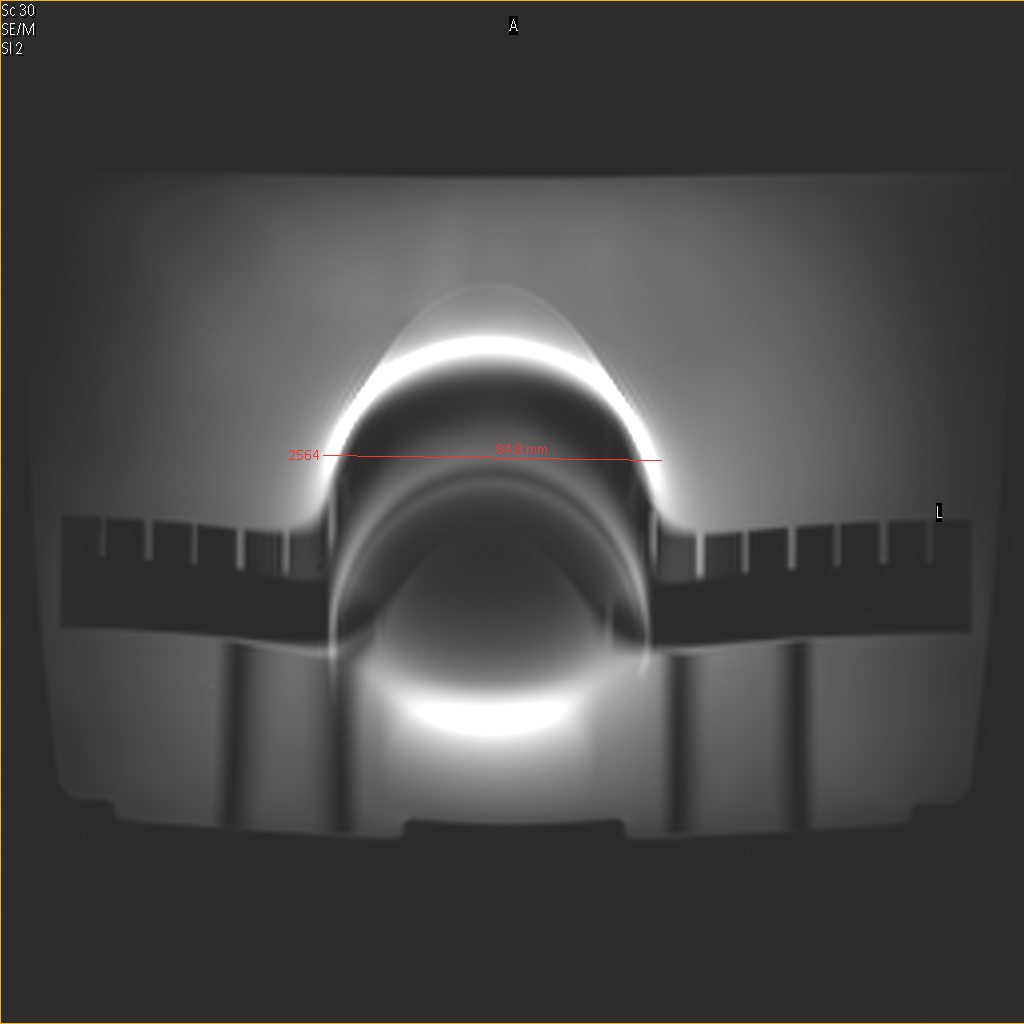

Supplement: S1 File — (ZIP) [file pone.0204220.s001.zip › artifacts/S14.png]

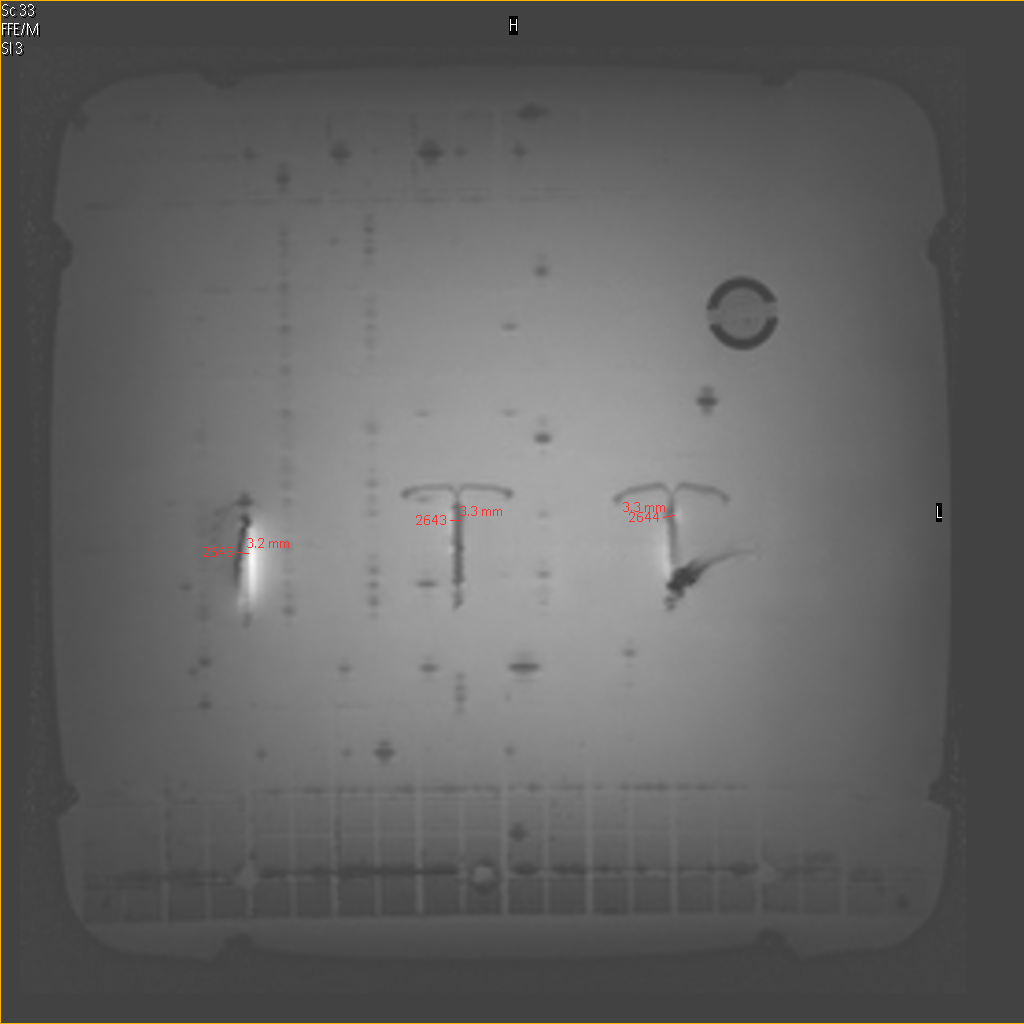

Supplement: S1 File — (ZIP) [file pone.0204220.s001.zip › artifacts/S15.png]

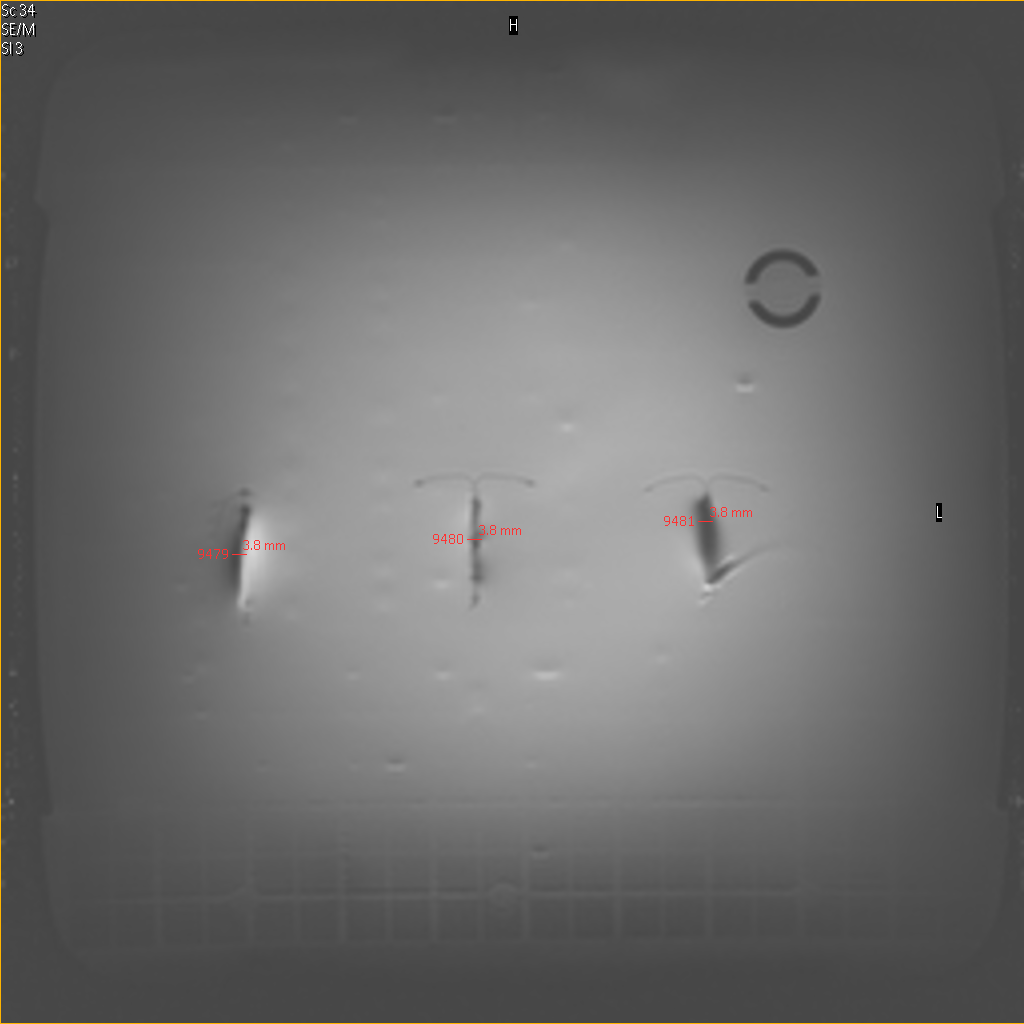

Supplement: S1 File — (ZIP) [file pone.0204220.s001.zip › artifacts/S16.png]

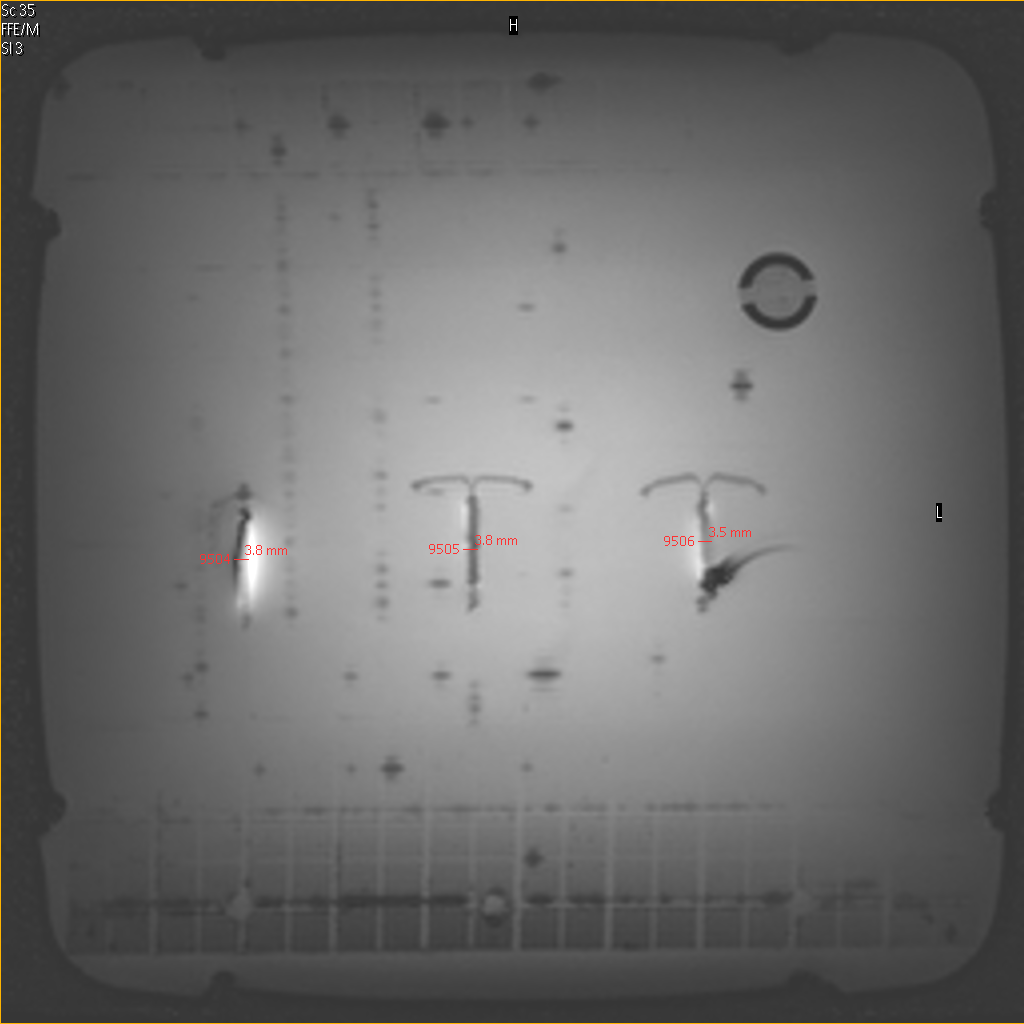

Supplement: S1 File — (ZIP) [file pone.0204220.s001.zip › artifacts/S17.png]

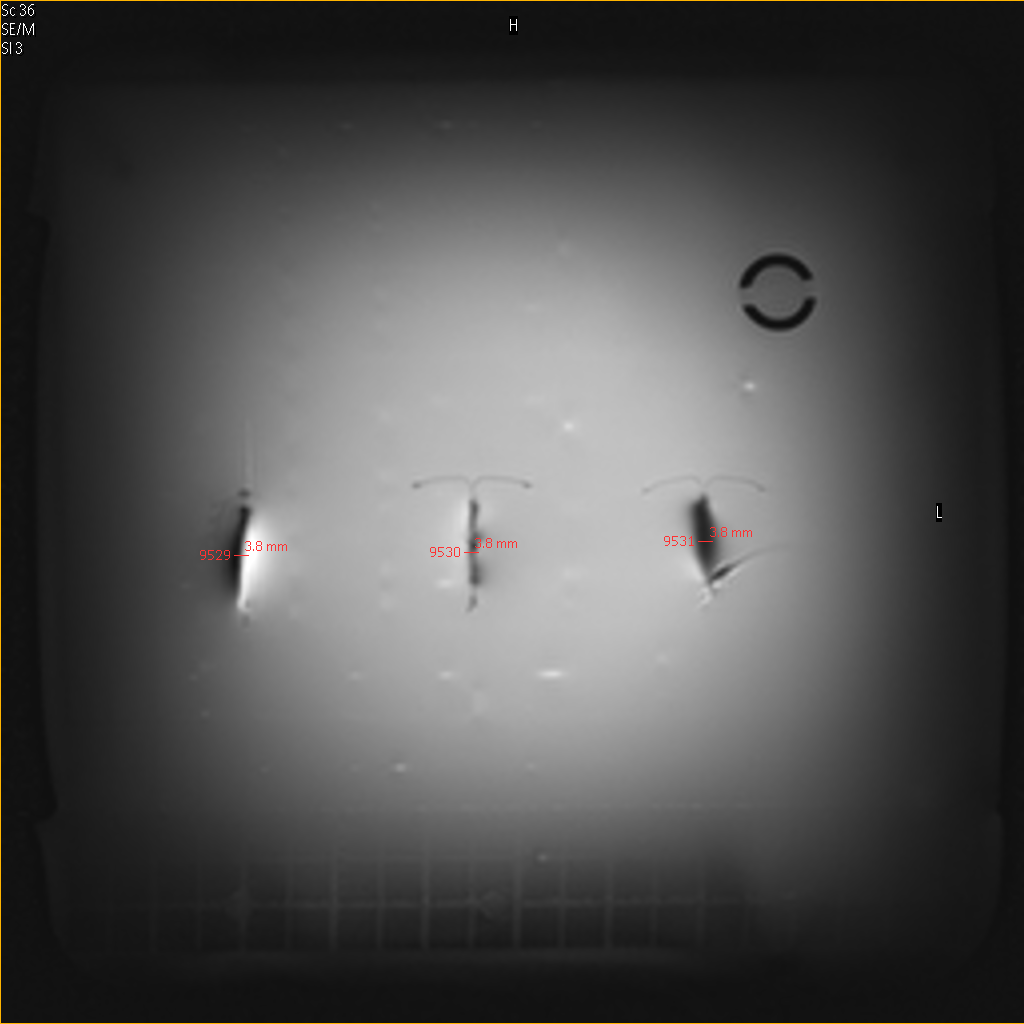

Supplement: S1 File — (ZIP) [file pone.0204220.s001.zip › artifacts/S18.png]

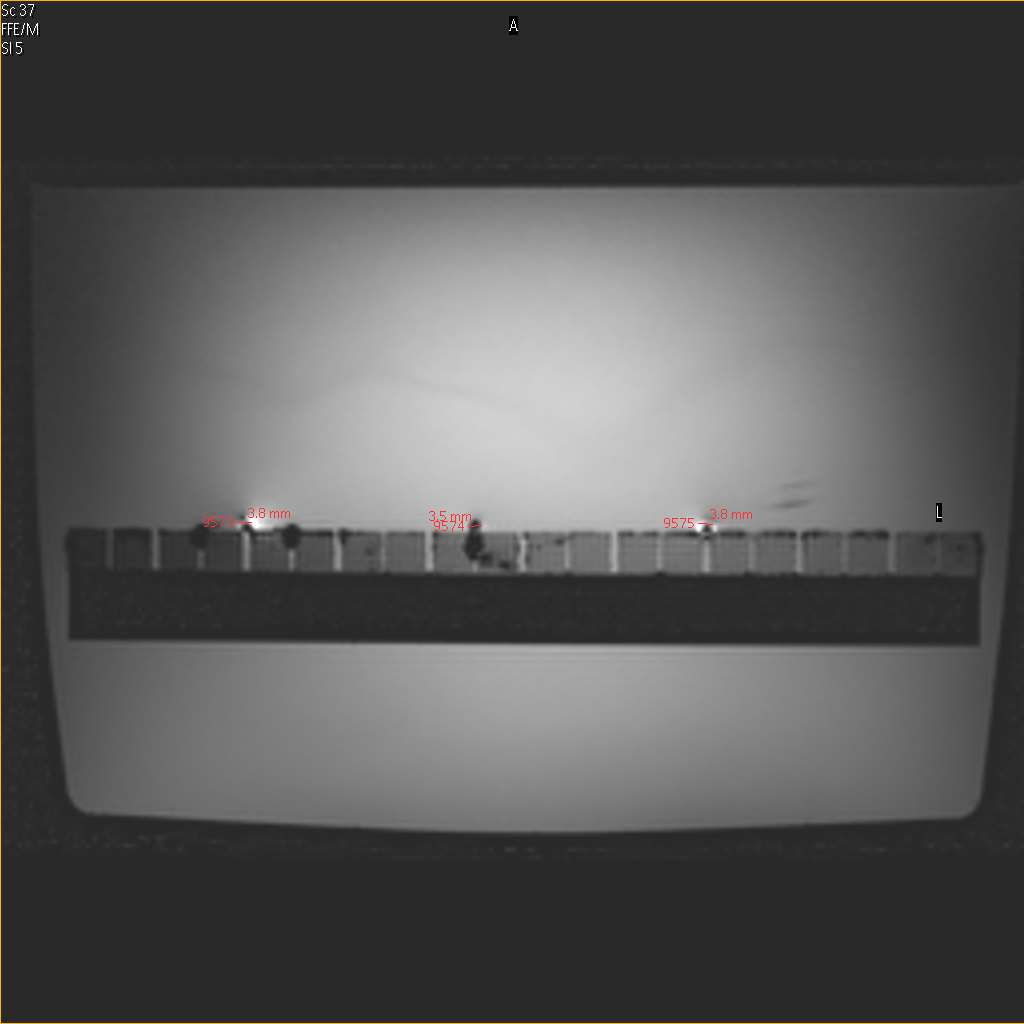

Supplement: S1 File — (ZIP) [file pone.0204220.s001.zip › artifacts/S19.png]

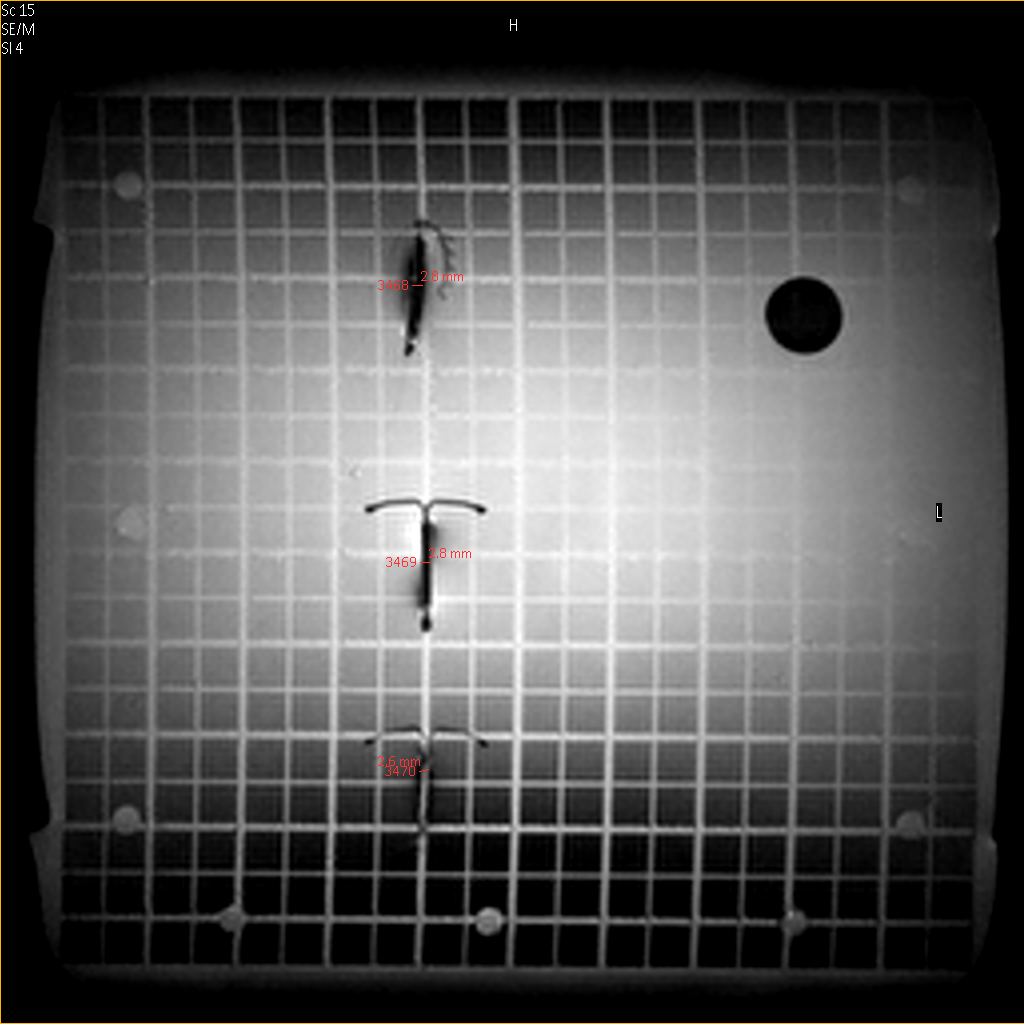

Supplement: S1 File — (ZIP) [file pone.0204220.s001.zip › artifacts/S2.png]

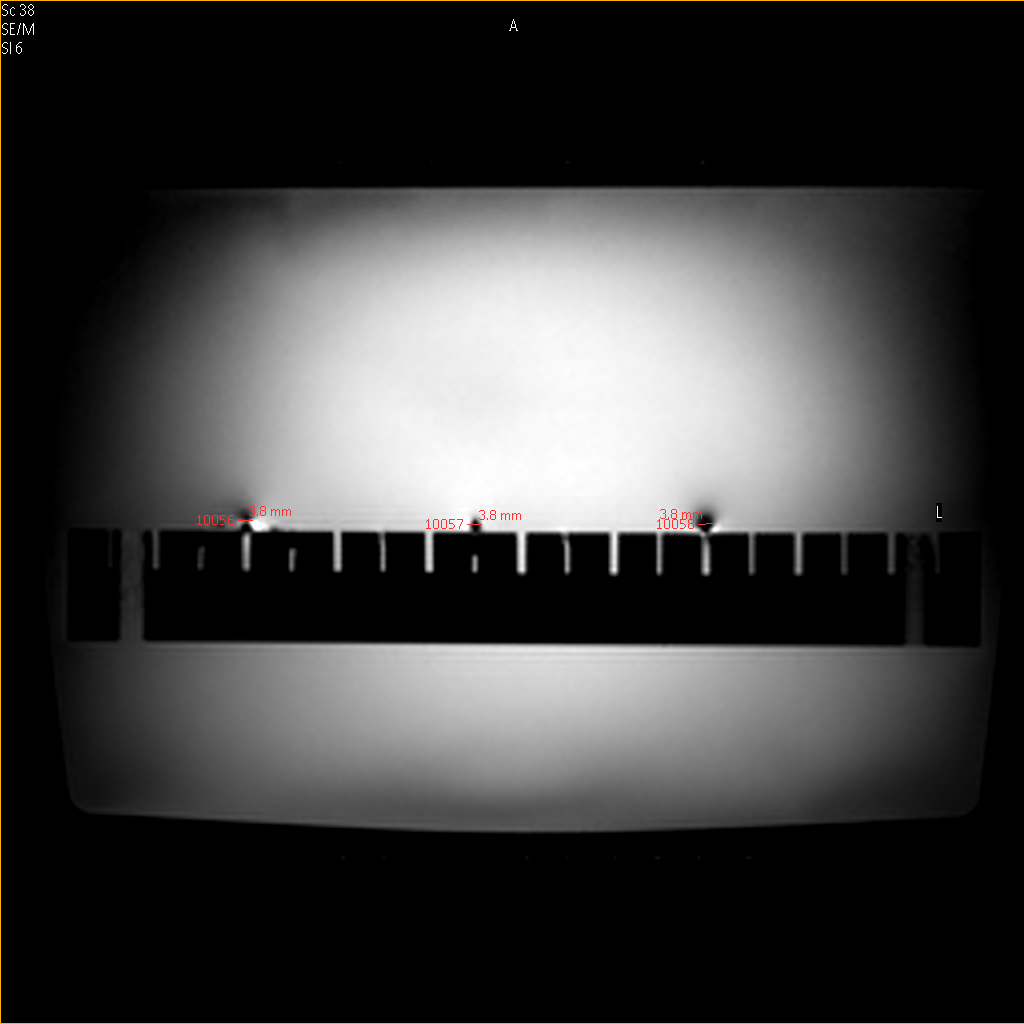

Supplement: S1 File — (ZIP) [file pone.0204220.s001.zip › artifacts/S20.png]

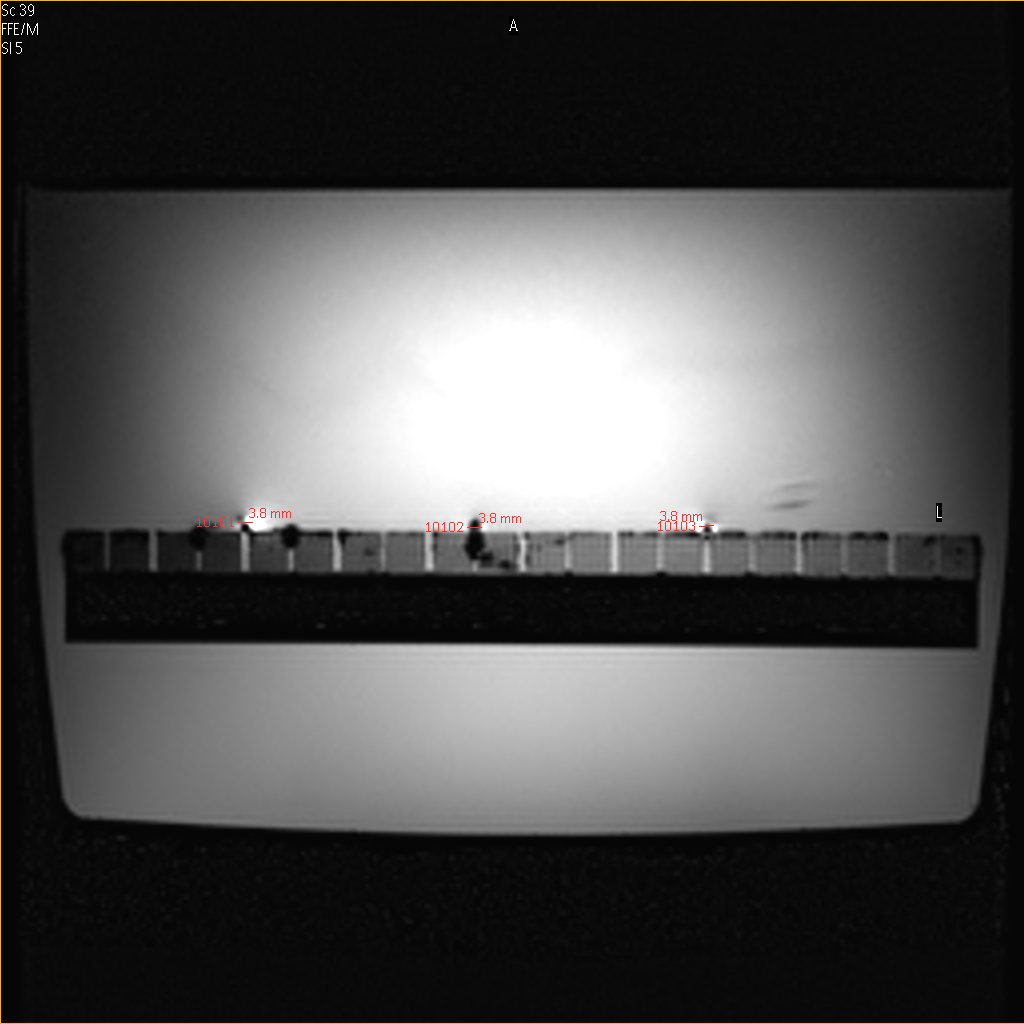

Supplement: S1 File — (ZIP) [file pone.0204220.s001.zip › artifacts/S21.png]

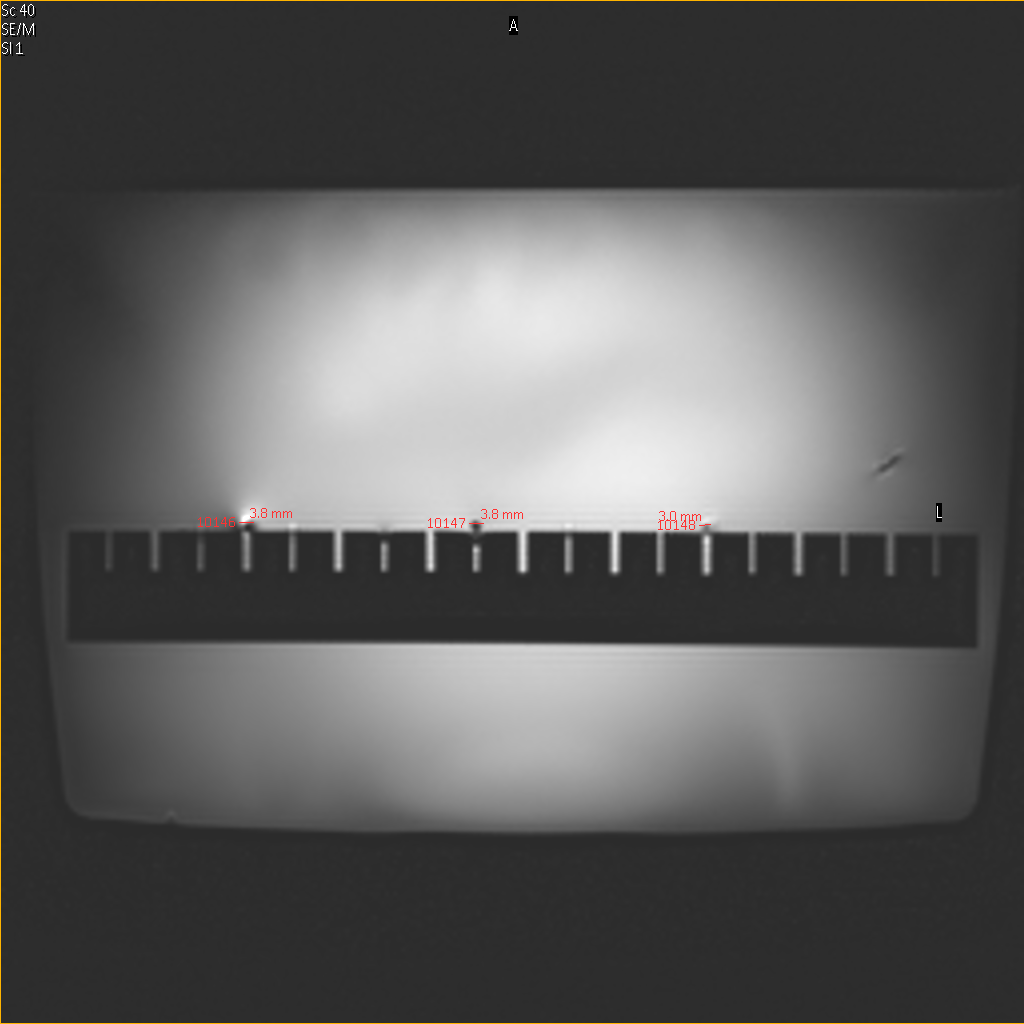

Supplement: S1 File — (ZIP) [file pone.0204220.s001.zip › artifacts/S22.png]

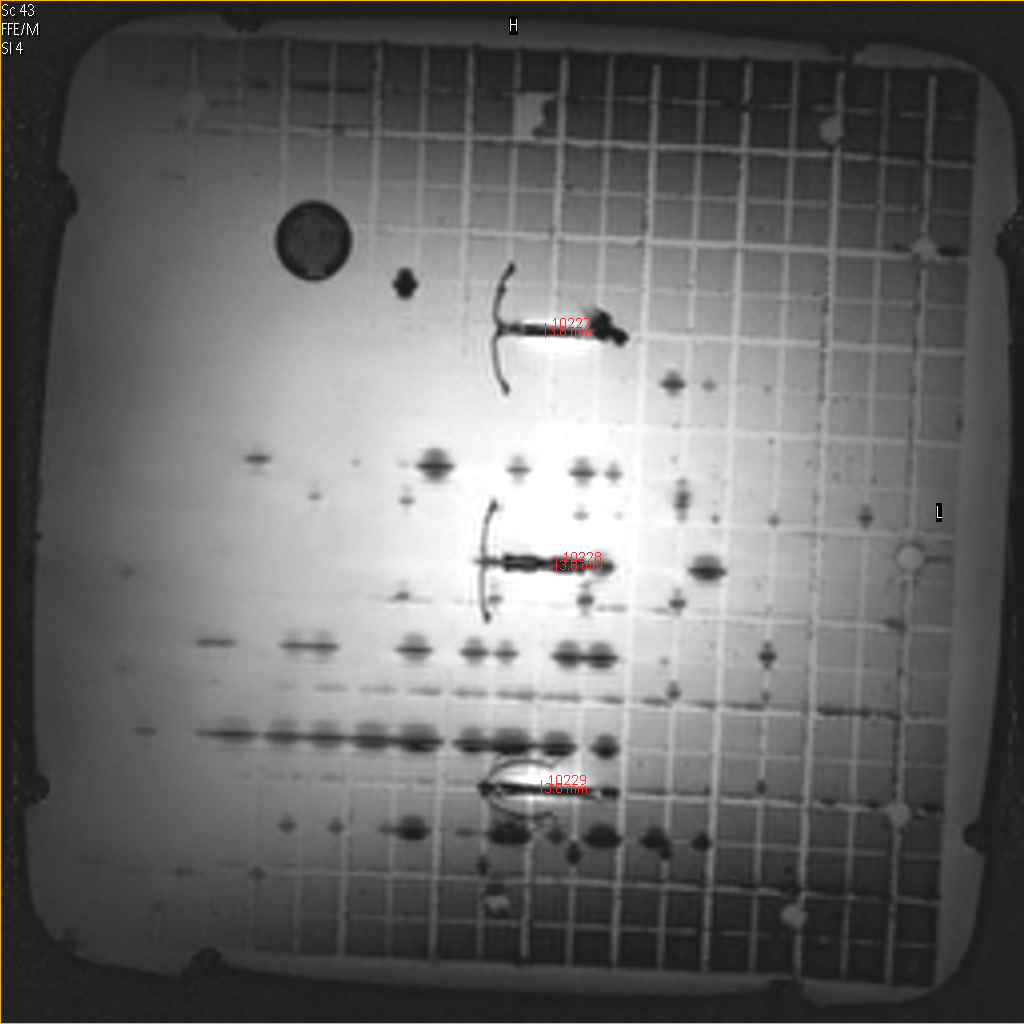

Supplement: S1 File — (ZIP) [file pone.0204220.s001.zip › artifacts/S23.png]

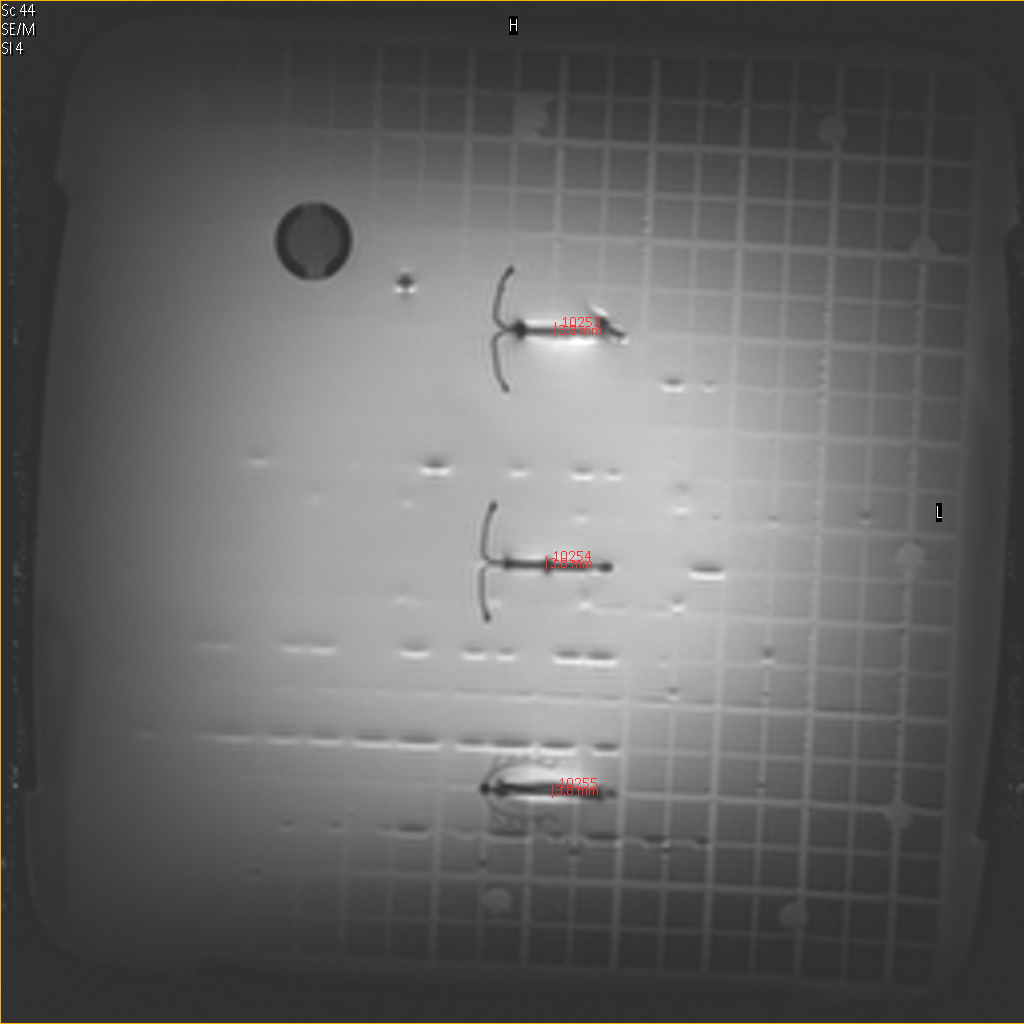

Supplement: S1 File — (ZIP) [file pone.0204220.s001.zip › artifacts/S24.png]

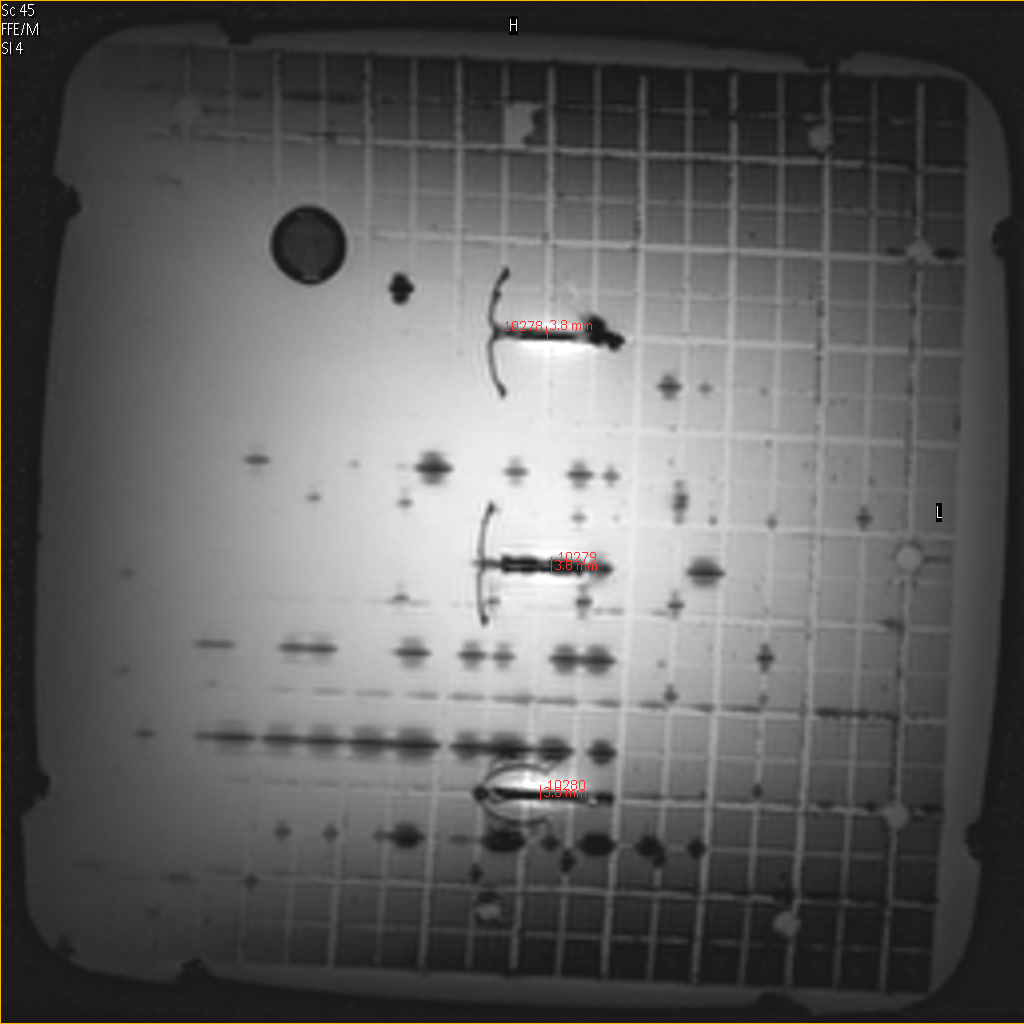

Supplement: S1 File — (ZIP) [file pone.0204220.s001.zip › artifacts/S25.png]

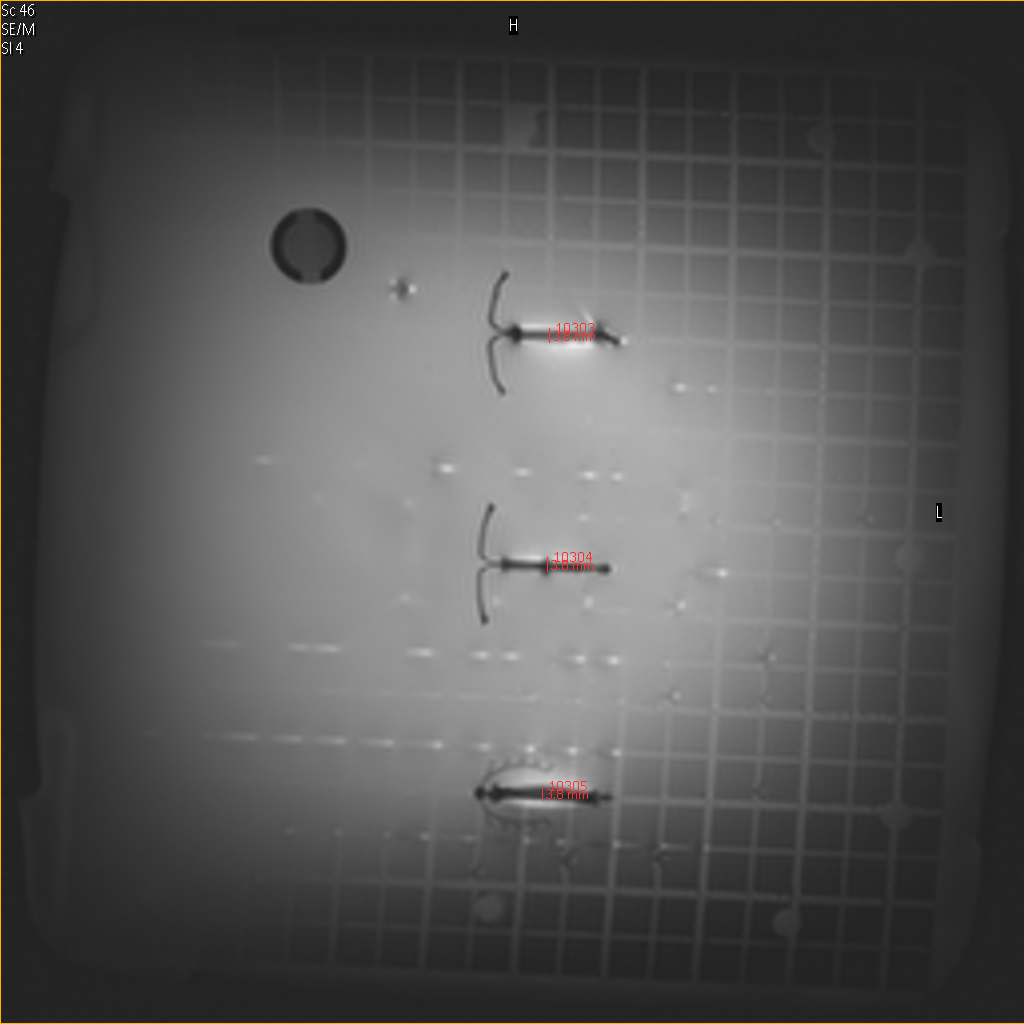

Supplement: S1 File — (ZIP) [file pone.0204220.s001.zip › artifacts/S26.png]

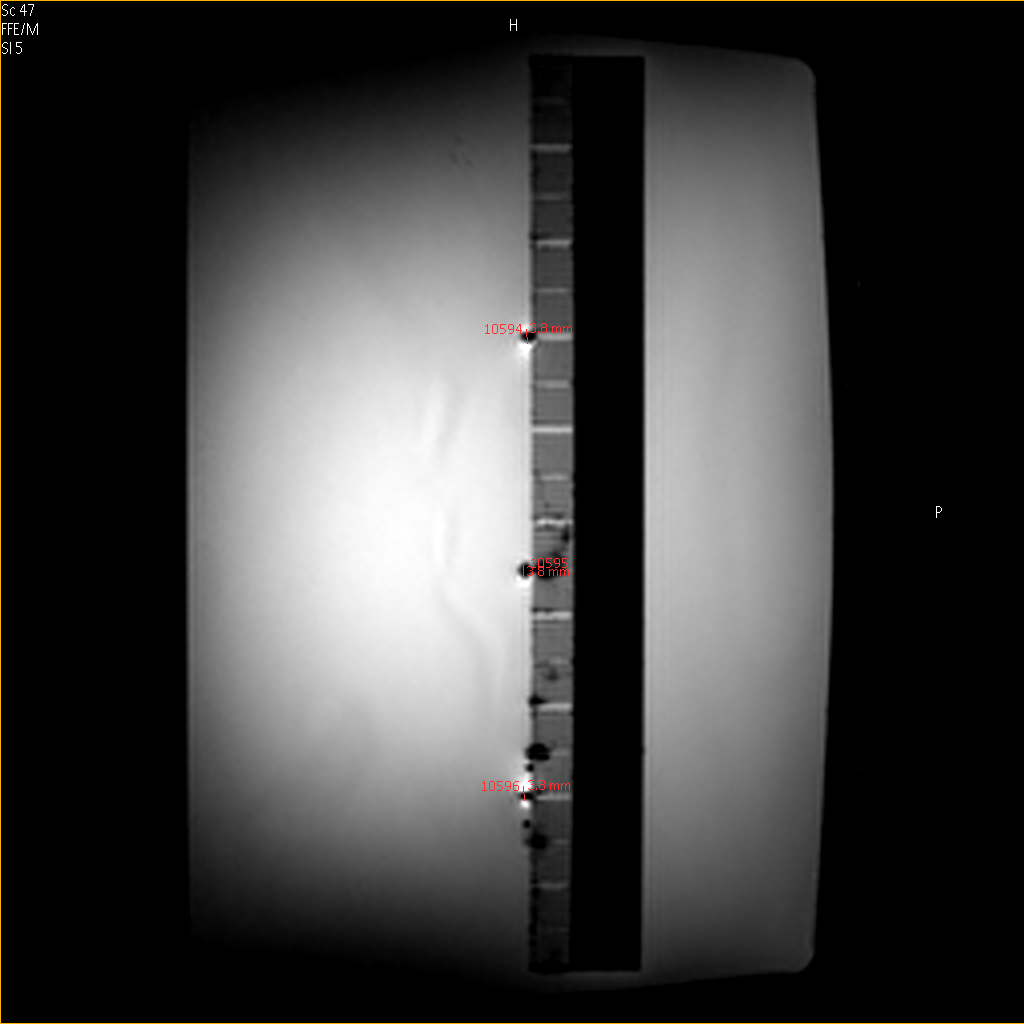

Supplement: S1 File — (ZIP) [file pone.0204220.s001.zip › artifacts/S27.png]

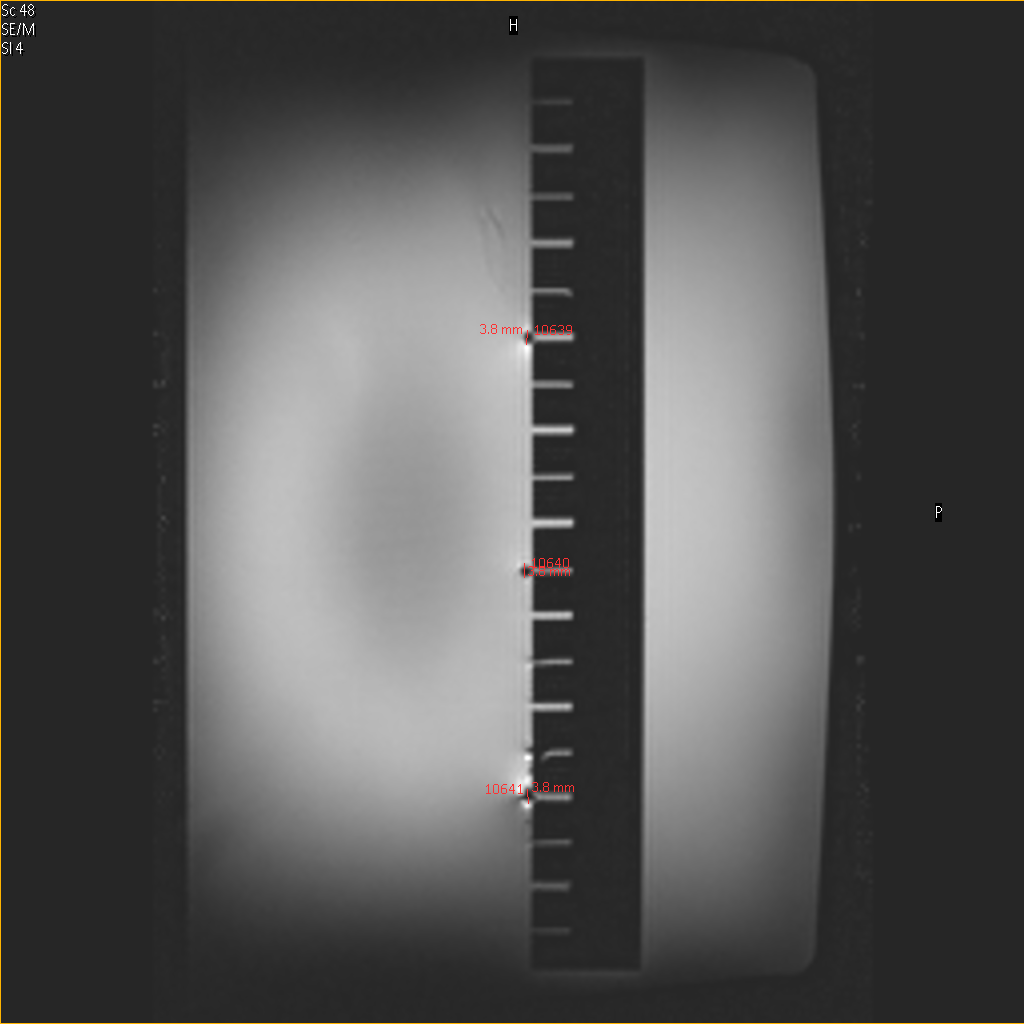

Supplement: S1 File — (ZIP) [file pone.0204220.s001.zip › artifacts/S28.png]

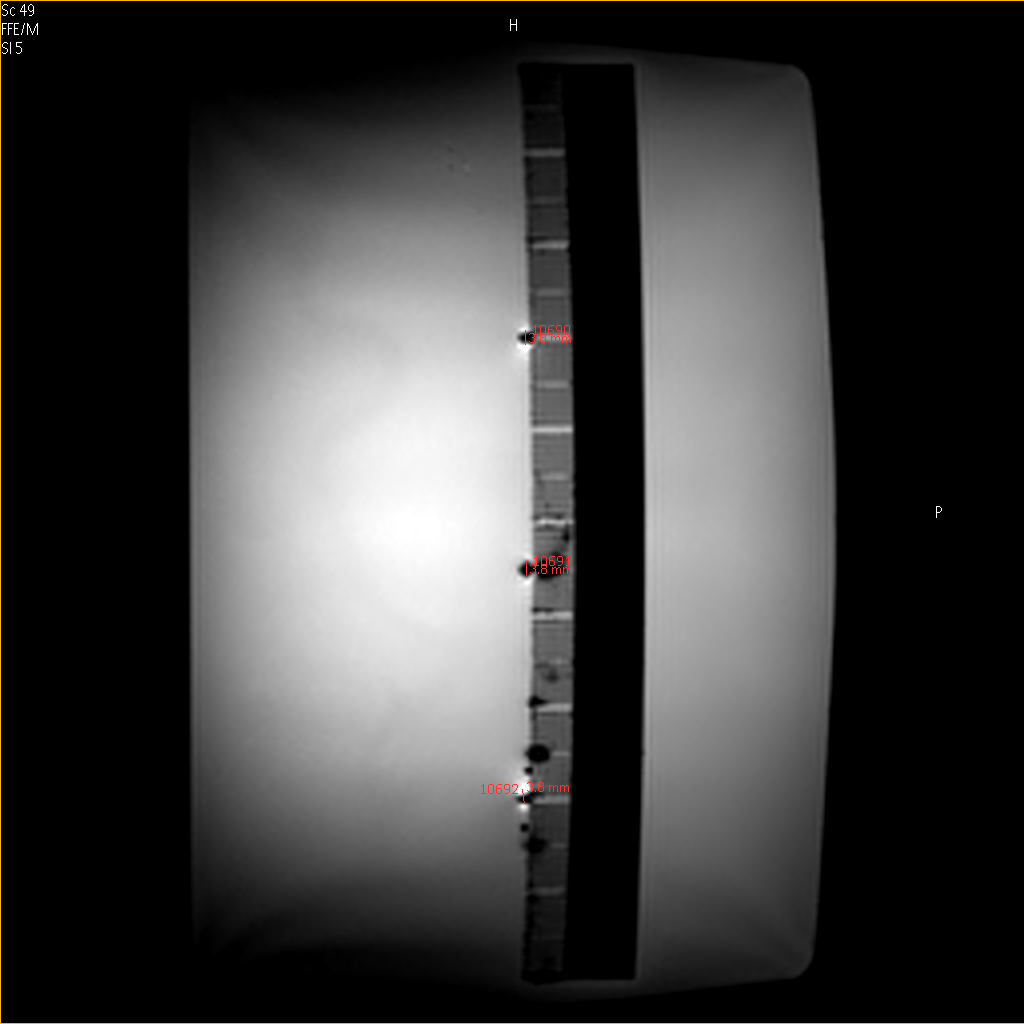

Supplement: S1 File — (ZIP) [file pone.0204220.s001.zip › artifacts/S29.png]

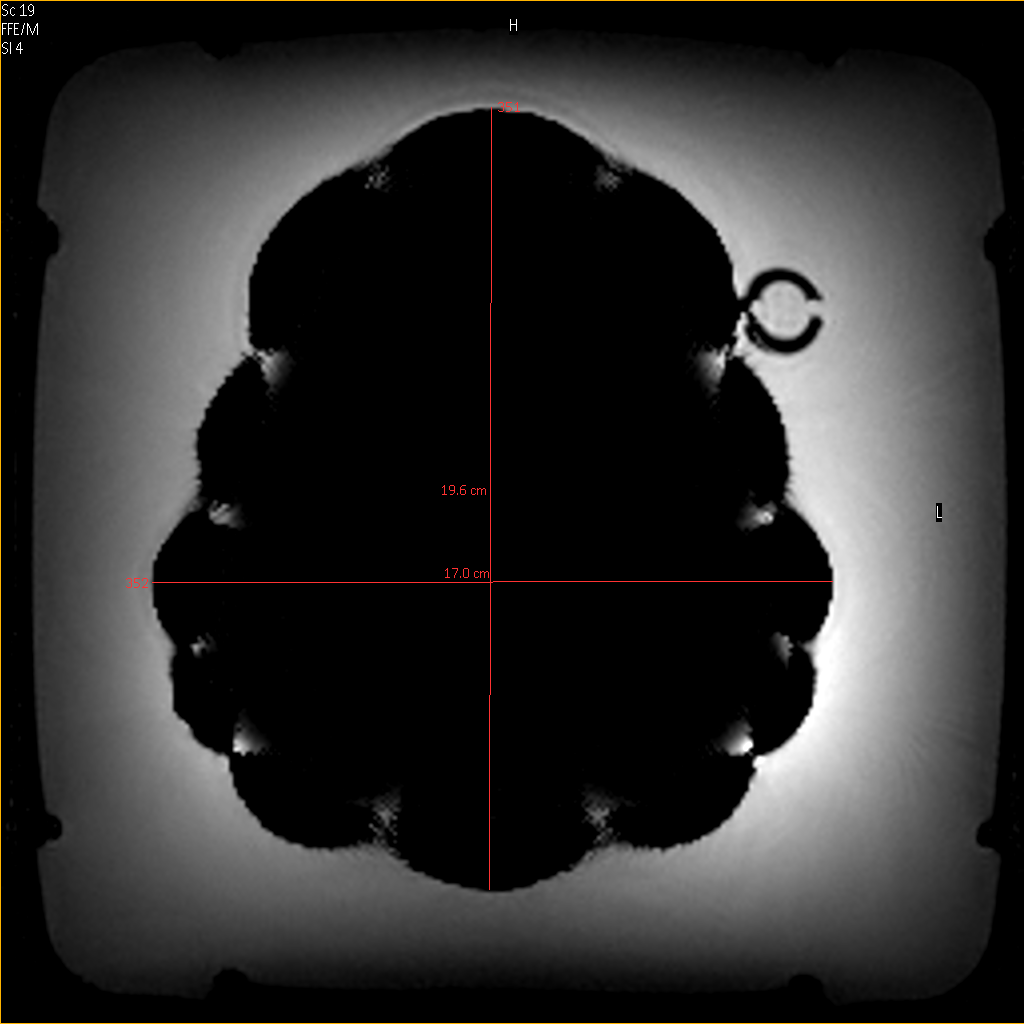

Supplement: S1 File — (ZIP) [file pone.0204220.s001.zip › artifacts/S3.png]

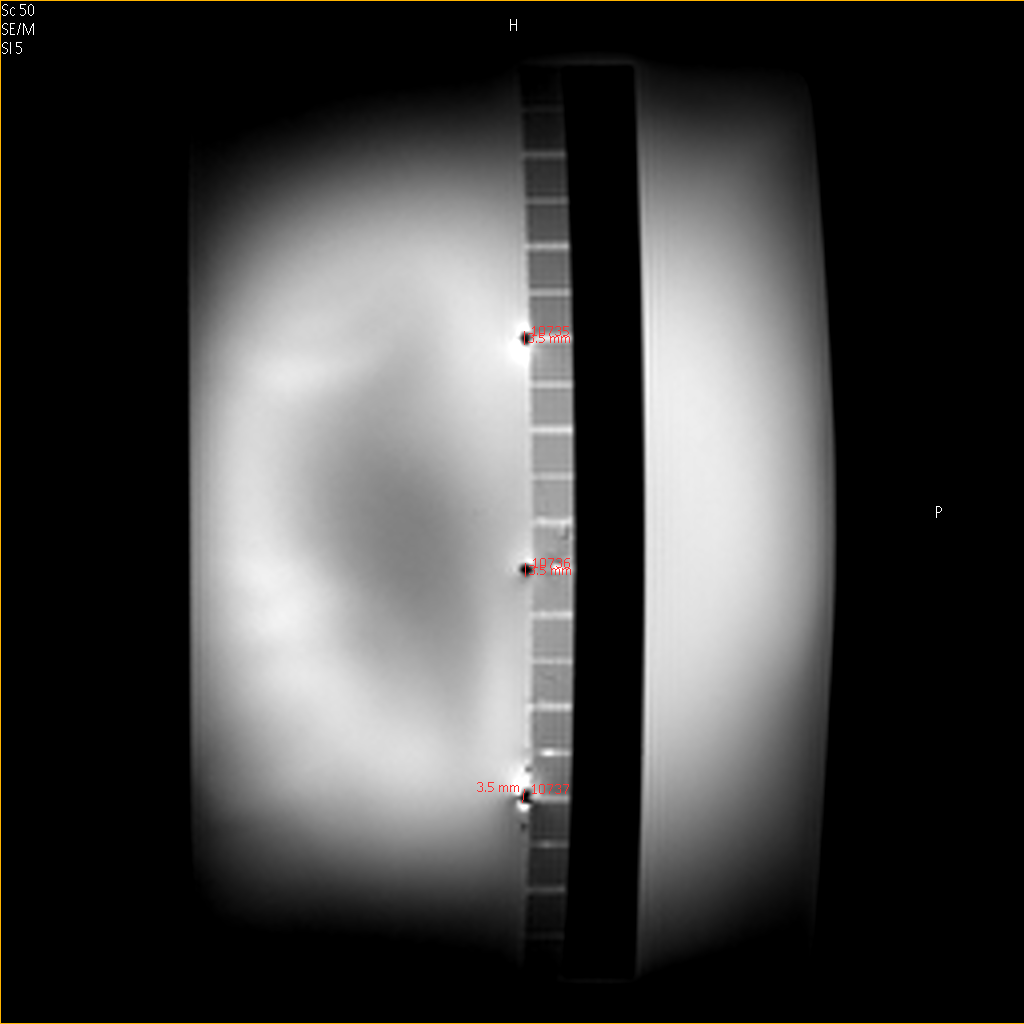

Supplement: S1 File — (ZIP) [file pone.0204220.s001.zip › artifacts/S30.png]

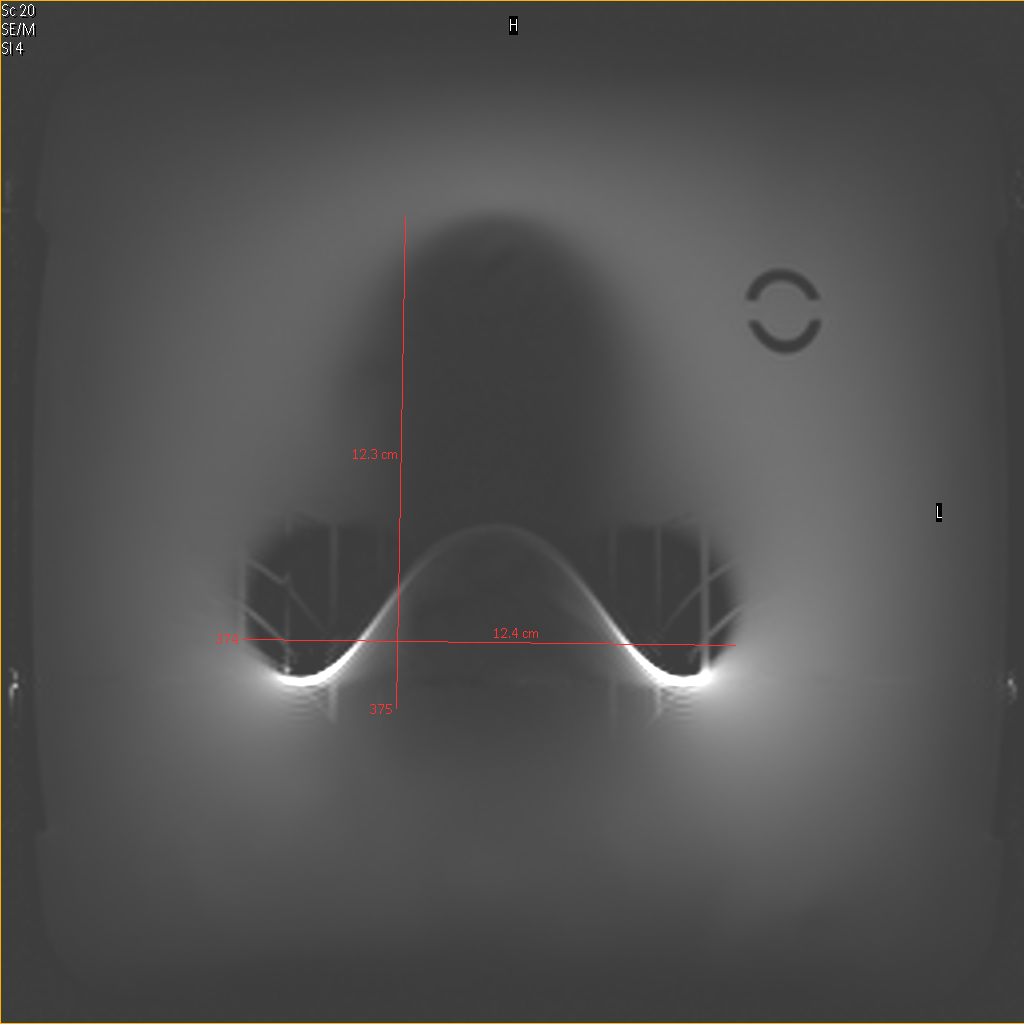

Supplement: S1 File — (ZIP) [file pone.0204220.s001.zip › artifacts/S4.png]

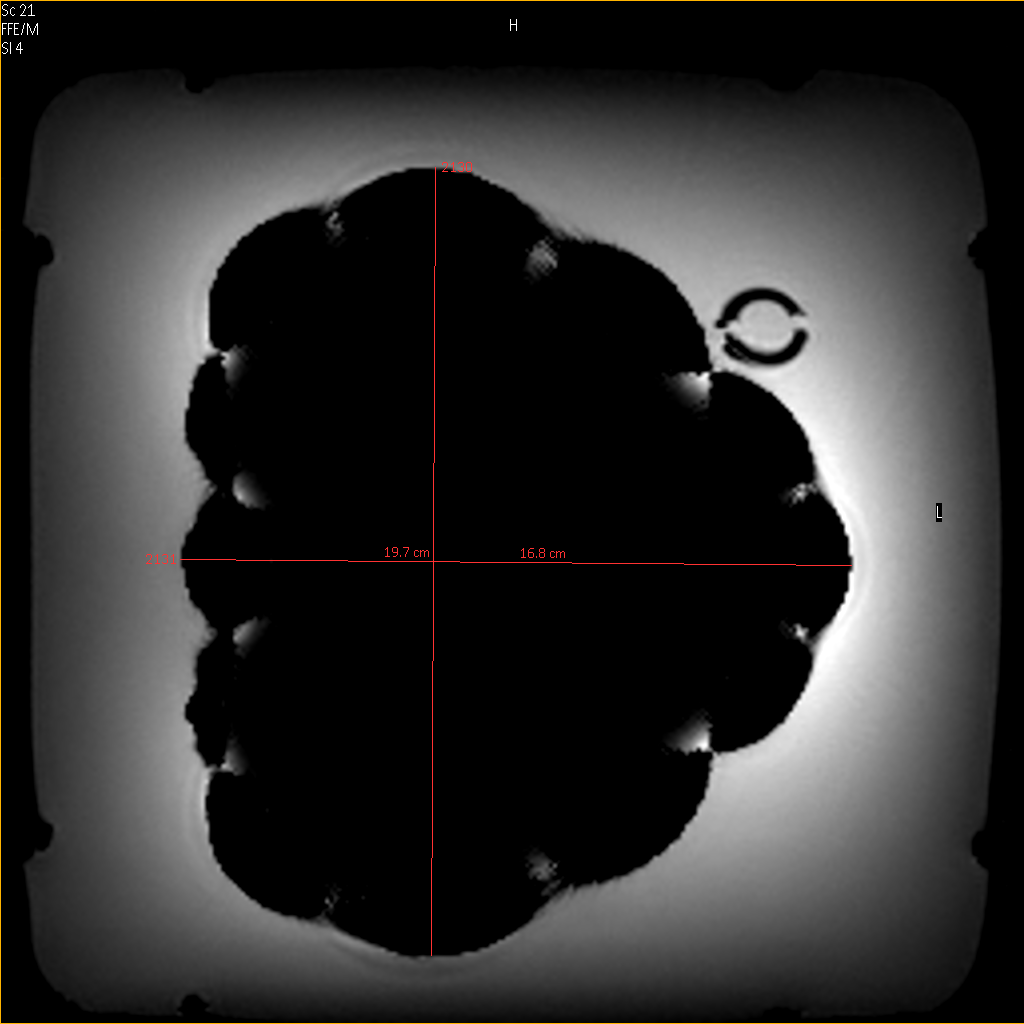

Supplement: S1 File — (ZIP) [file pone.0204220.s001.zip › artifacts/S5.png]

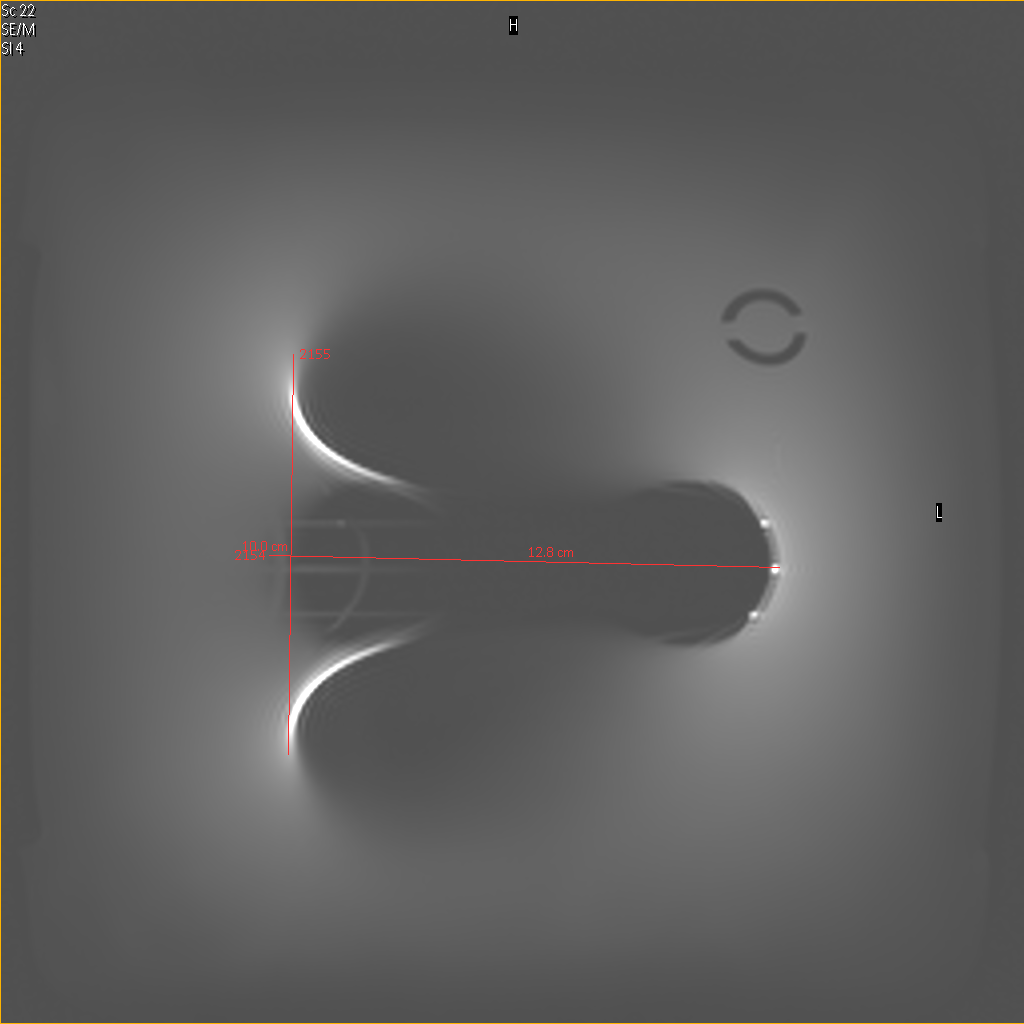

Supplement: S1 File — (ZIP) [file pone.0204220.s001.zip › artifacts/S6.png]

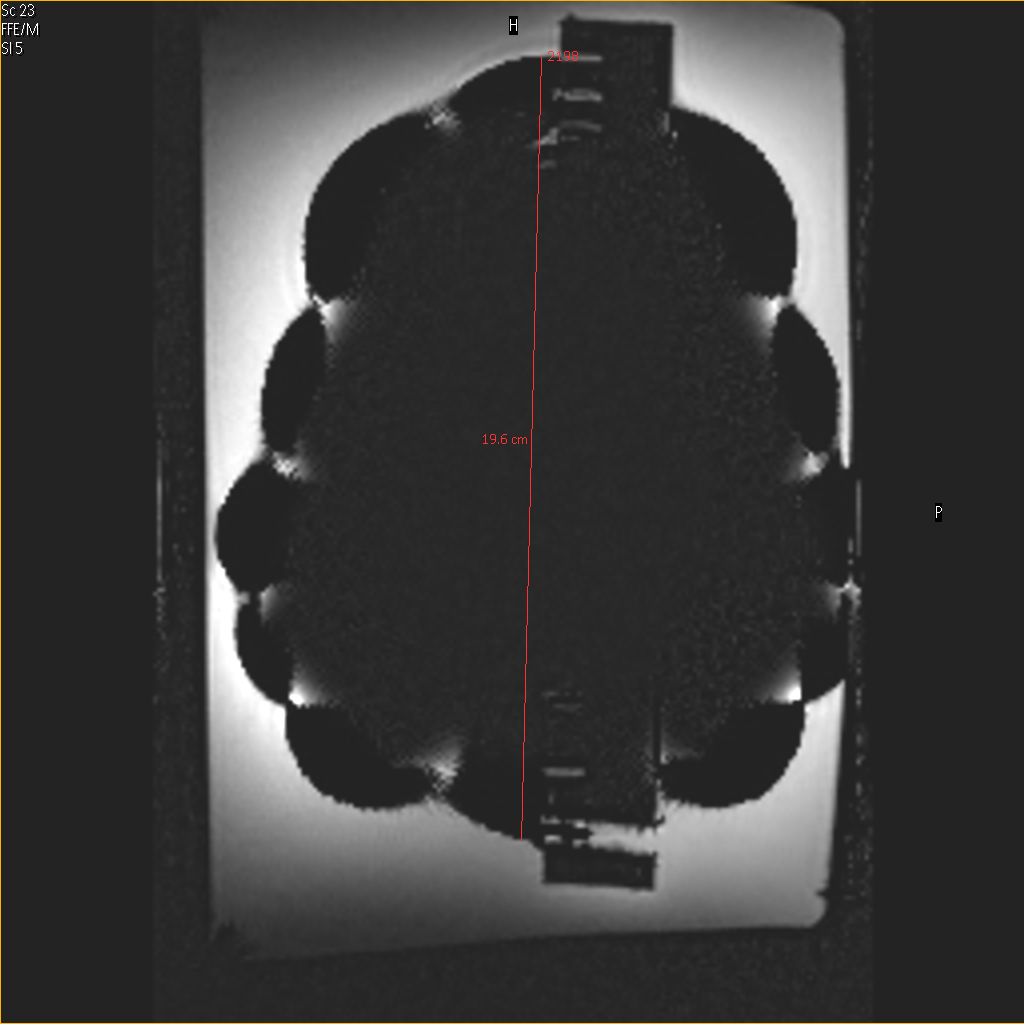

Supplement: S1 File — (ZIP) [file pone.0204220.s001.zip › artifacts/S7.png]

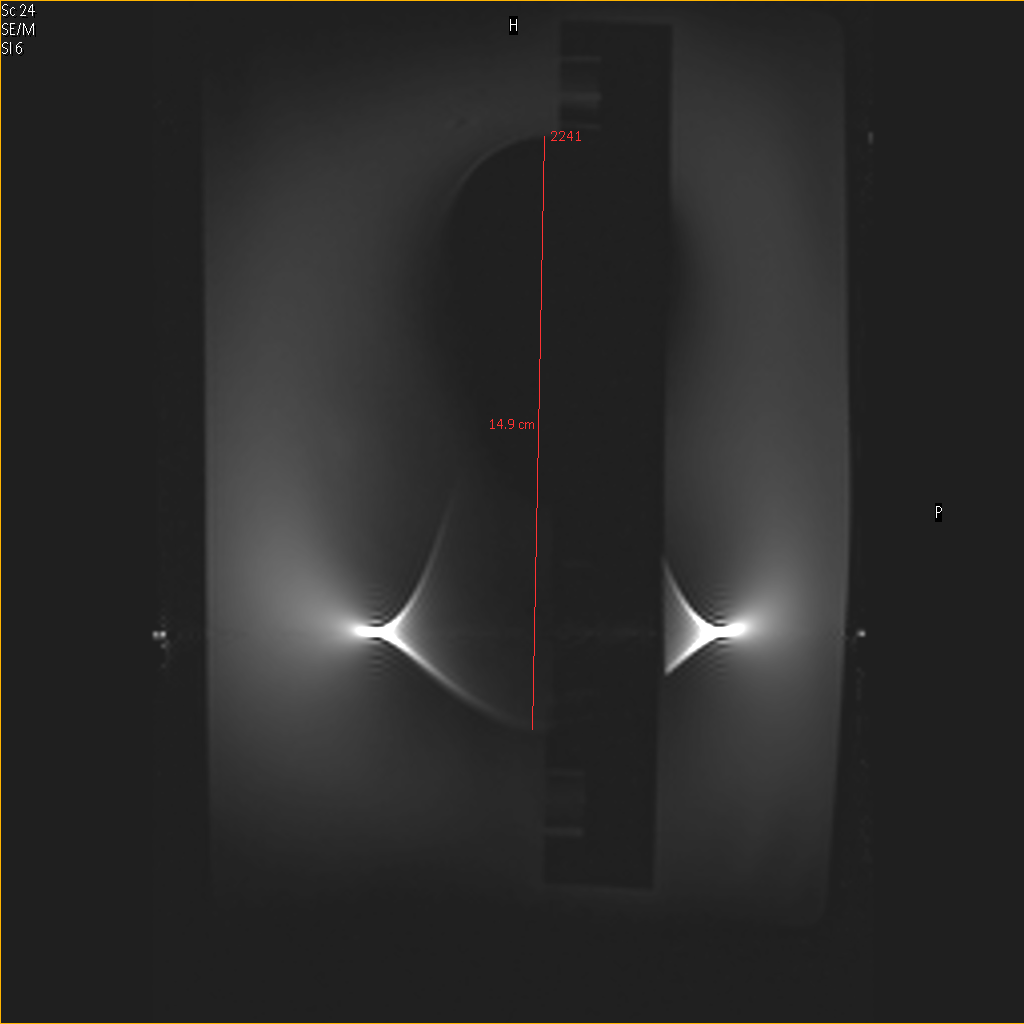

Supplement: S1 File — (ZIP) [file pone.0204220.s001.zip › artifacts/S8.png]

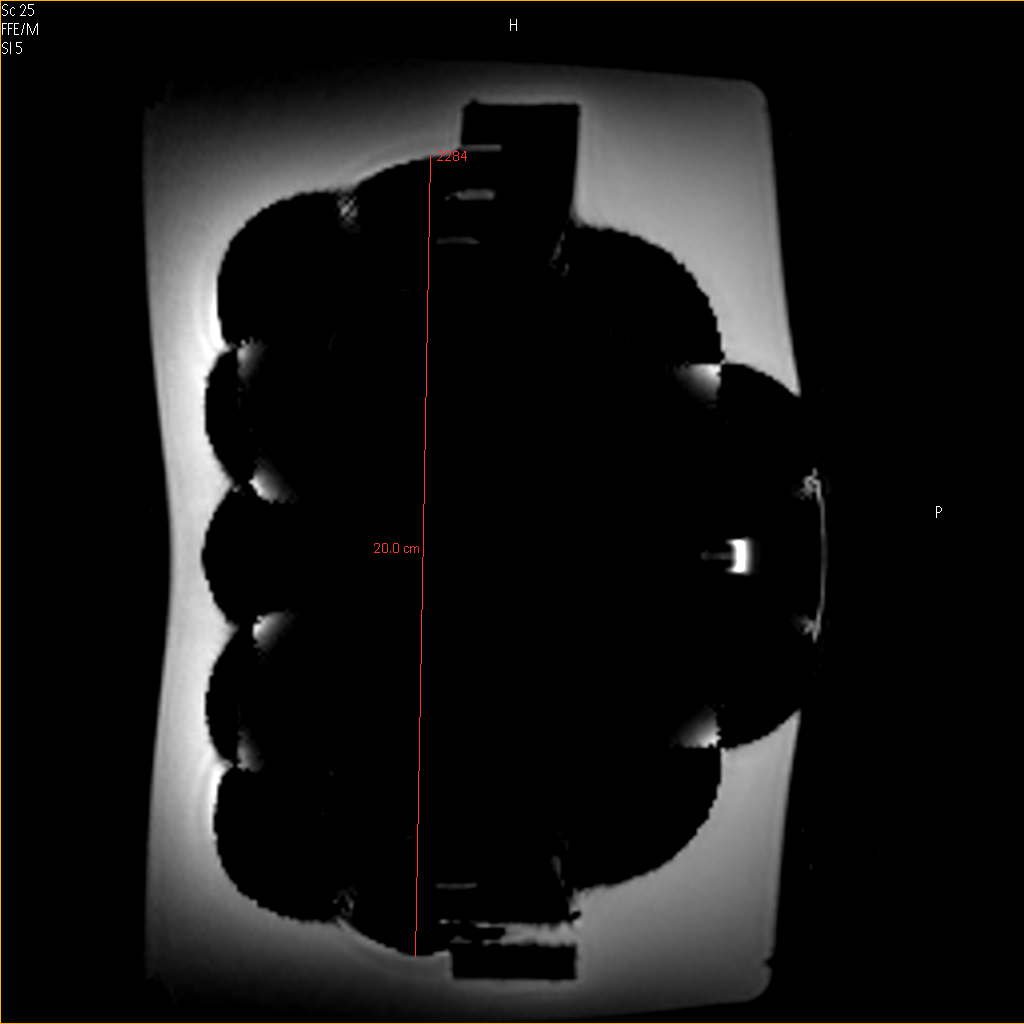

Supplement: S1 File — (ZIP) [file pone.0204220.s001.zip › artifacts/S9.png]

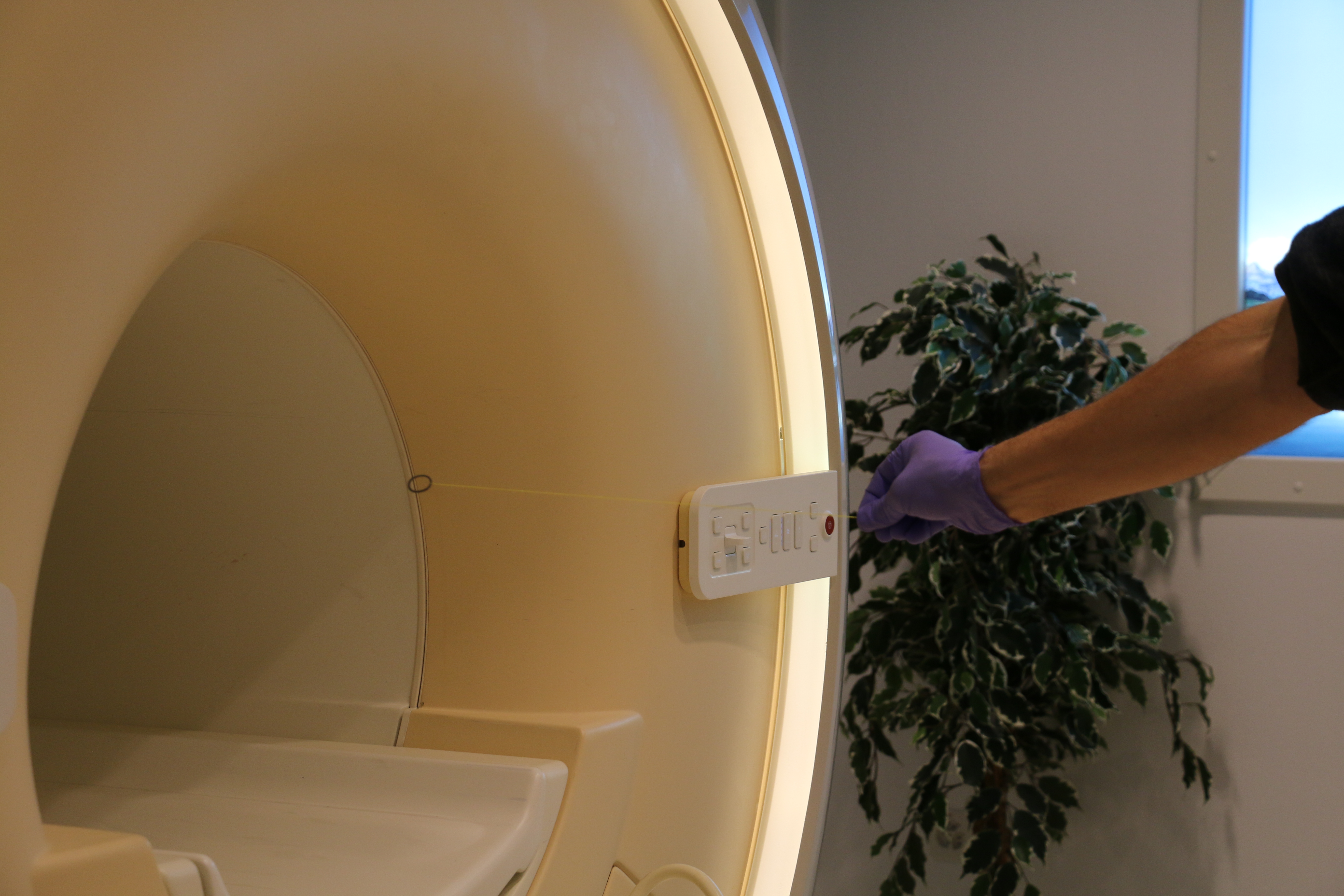

Supplement: S1 File — (ZIP) [file pone.0204220.s001.zip › force/S31.JPG]

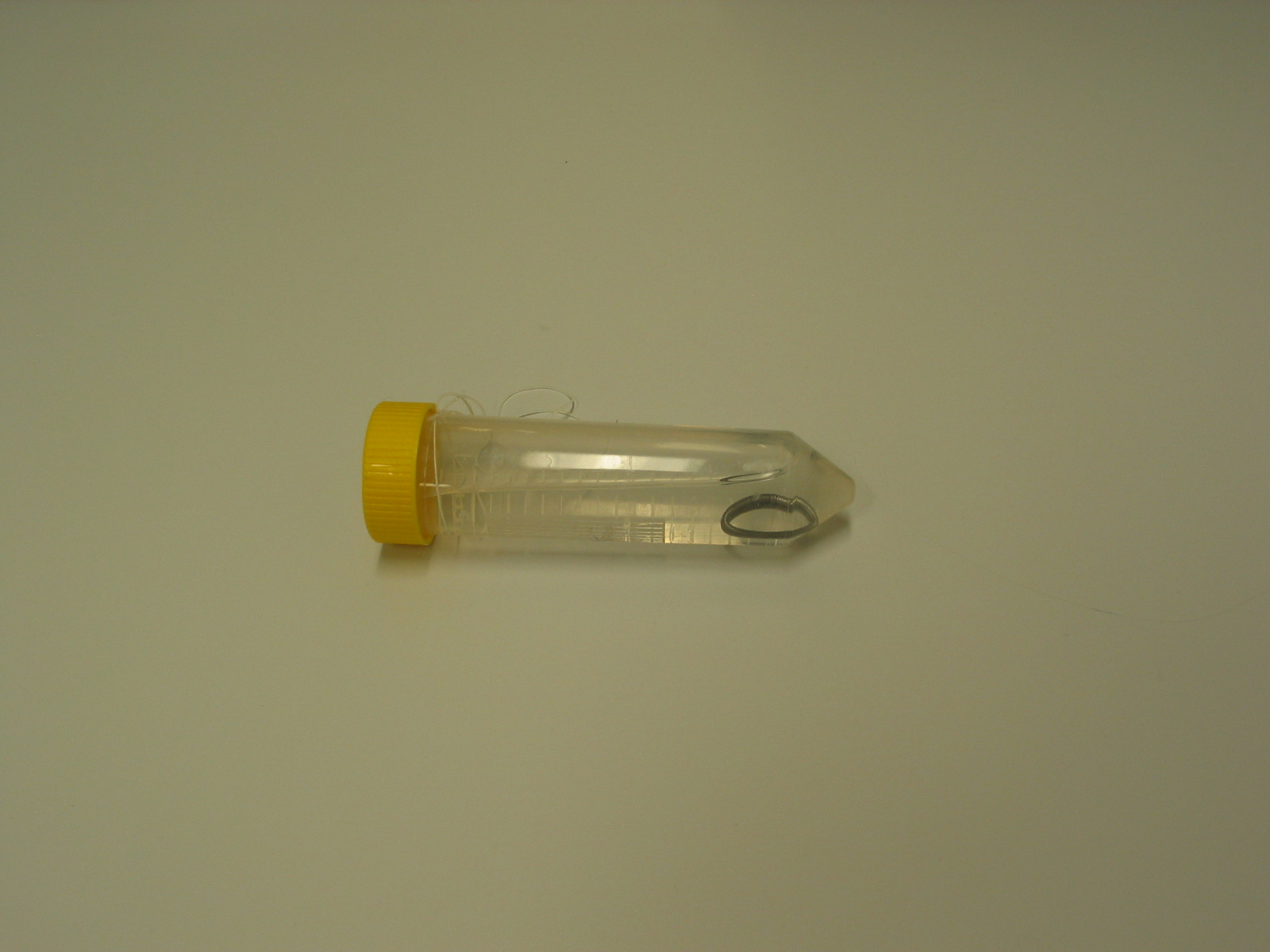

Supplement: S1 File — (ZIP) [file pone.0204220.s001.zip › force/S32.JPG]

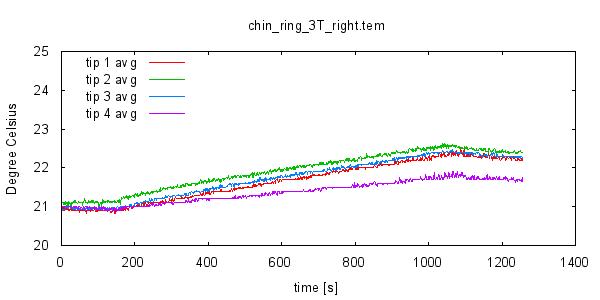

Supplement: S1 File — (ZIP) [file pone.0204220.s001.zip › rf_heating/chin_ring_3T_right.gif]

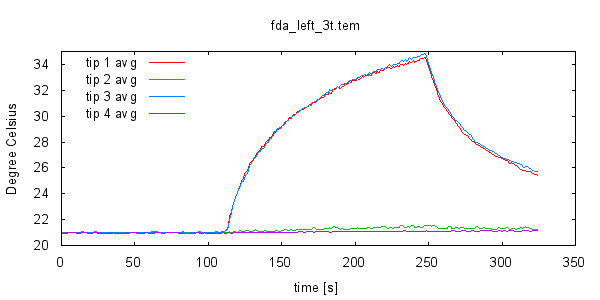

Supplement: S1 File — (ZIP) [file pone.0204220.s001.zip › rf_heating/fda_left_3t.gif]

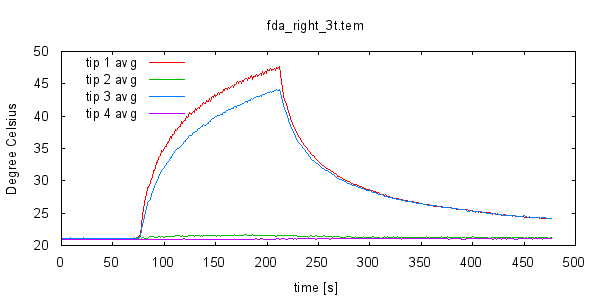

Supplement: S1 File — (ZIP) [file pone.0204220.s001.zip › rf_heating/fda_right_3t.gif]

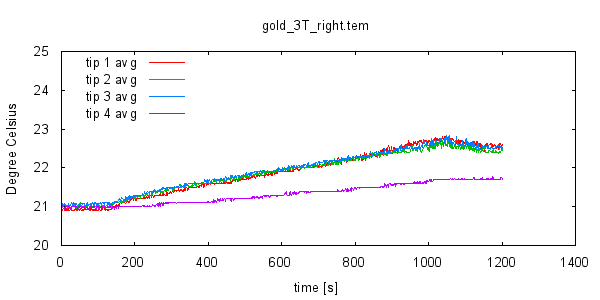

Supplement: S1 File — (ZIP) [file pone.0204220.s001.zip › rf_heating/gold_3T_right.gif]

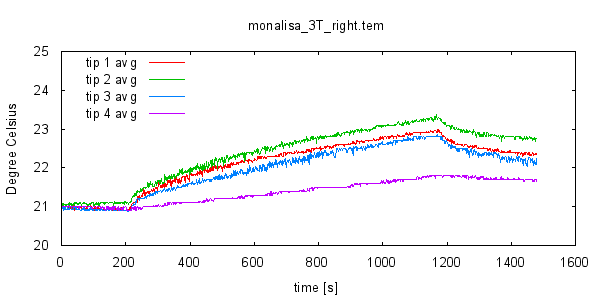

Supplement: S1 File — (ZIP) [file pone.0204220.s001.zip › rf_heating/monalisa_3T_right.gif]

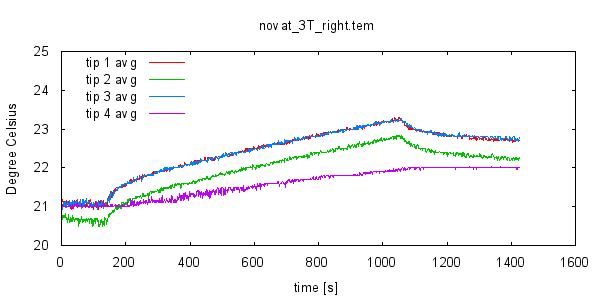

Supplement: S1 File — (ZIP) [file pone.0204220.s001.zip › rf_heating/novat_3T_right.gif]

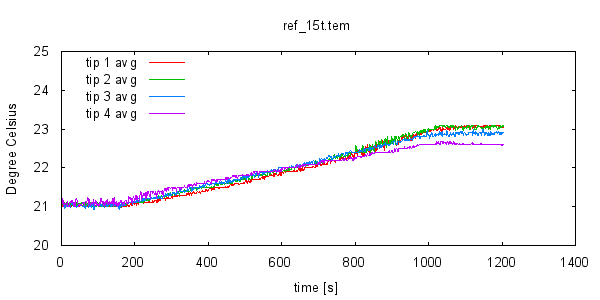

Supplement: S1 File — (ZIP) [file pone.0204220.s001.zip › rf_heating/ref_15t.gif]

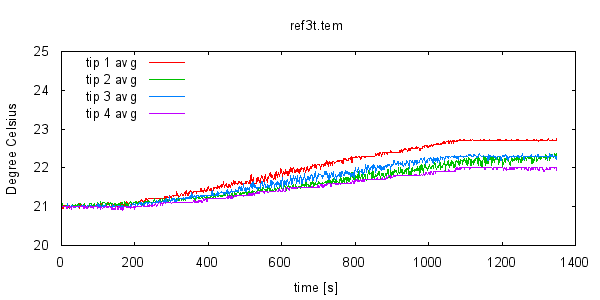

Supplement: S1 File — (ZIP) [file pone.0204220.s001.zip › rf_heating/ref3t.gif]

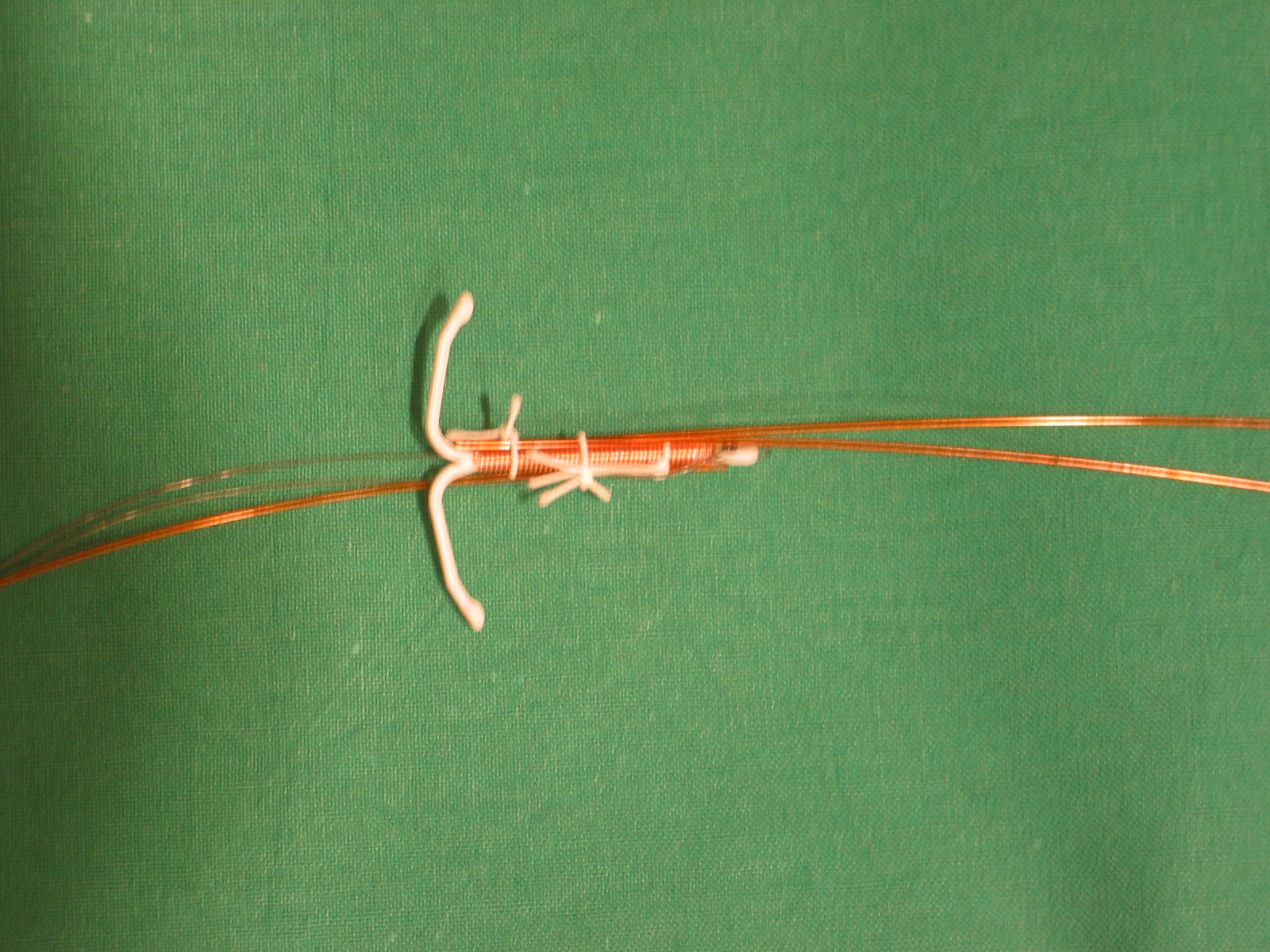

Supplement: S1 File — (ZIP) [file pone.0204220.s001.zip › rf_heating/S33.JPG]

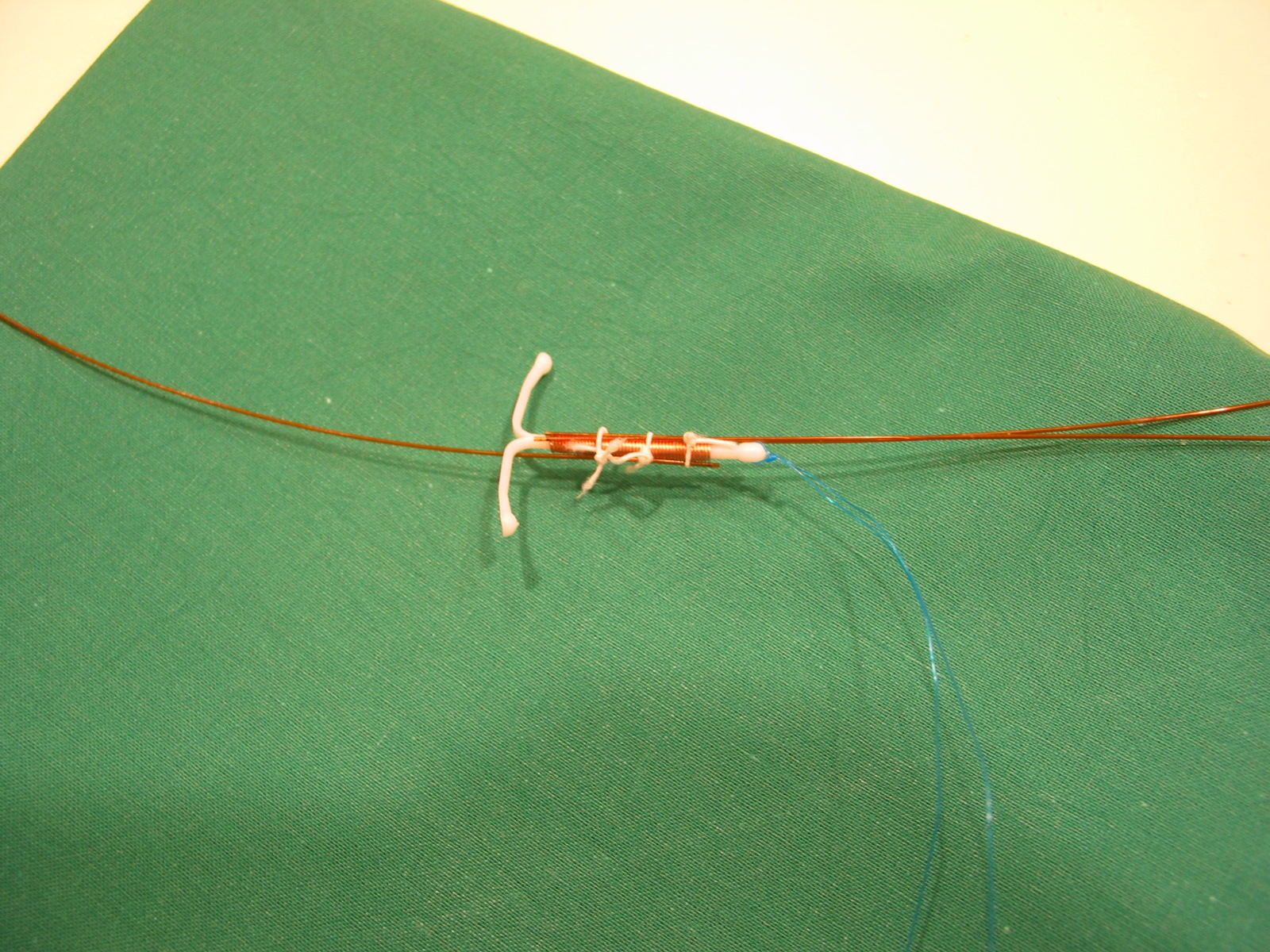

Supplement: S1 File — (ZIP) [file pone.0204220.s001.zip › rf_heating/S34.JPG]

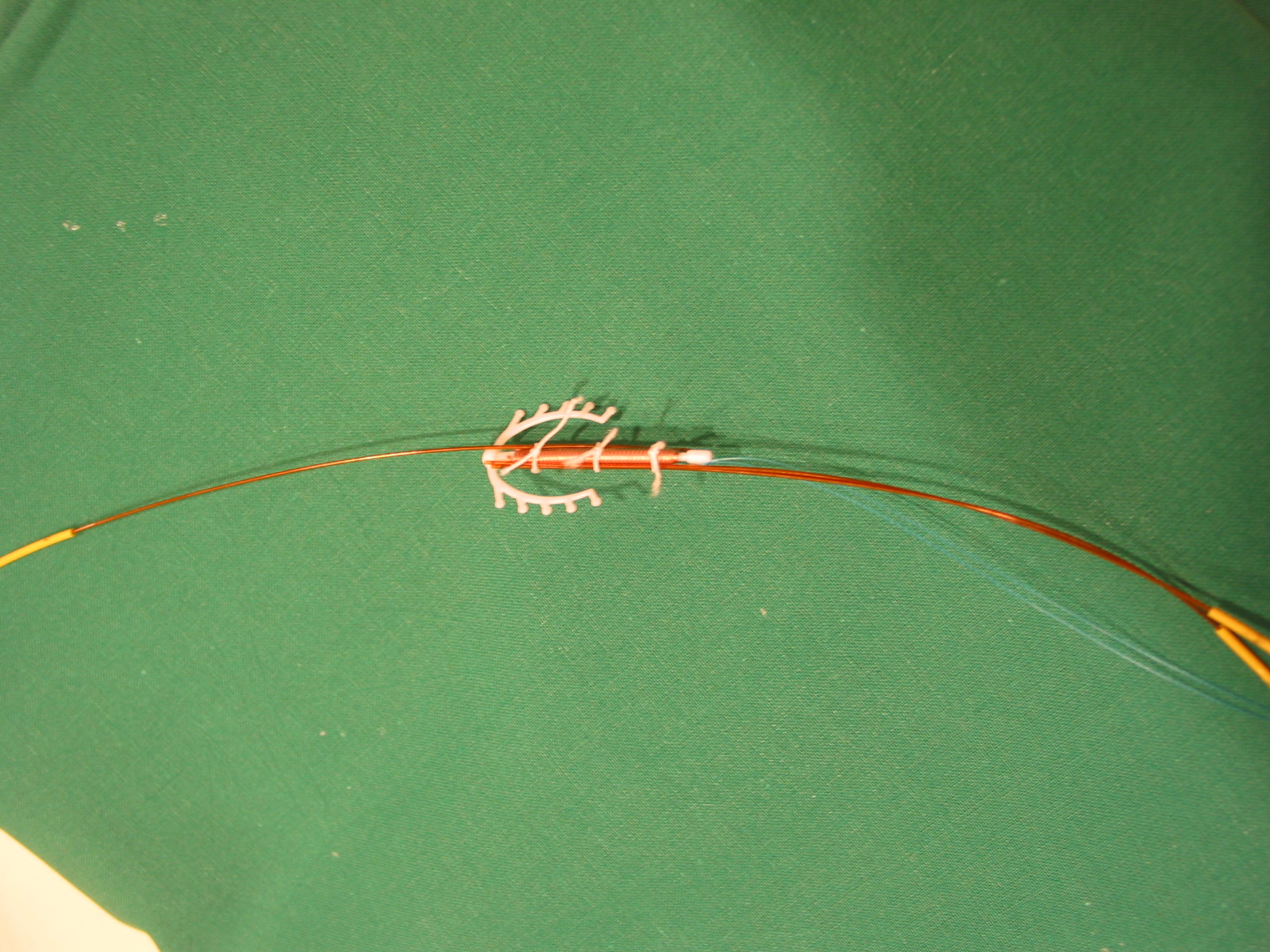

Supplement: S1 File — (ZIP) [file pone.0204220.s001.zip › rf_heating/S35.JPG]

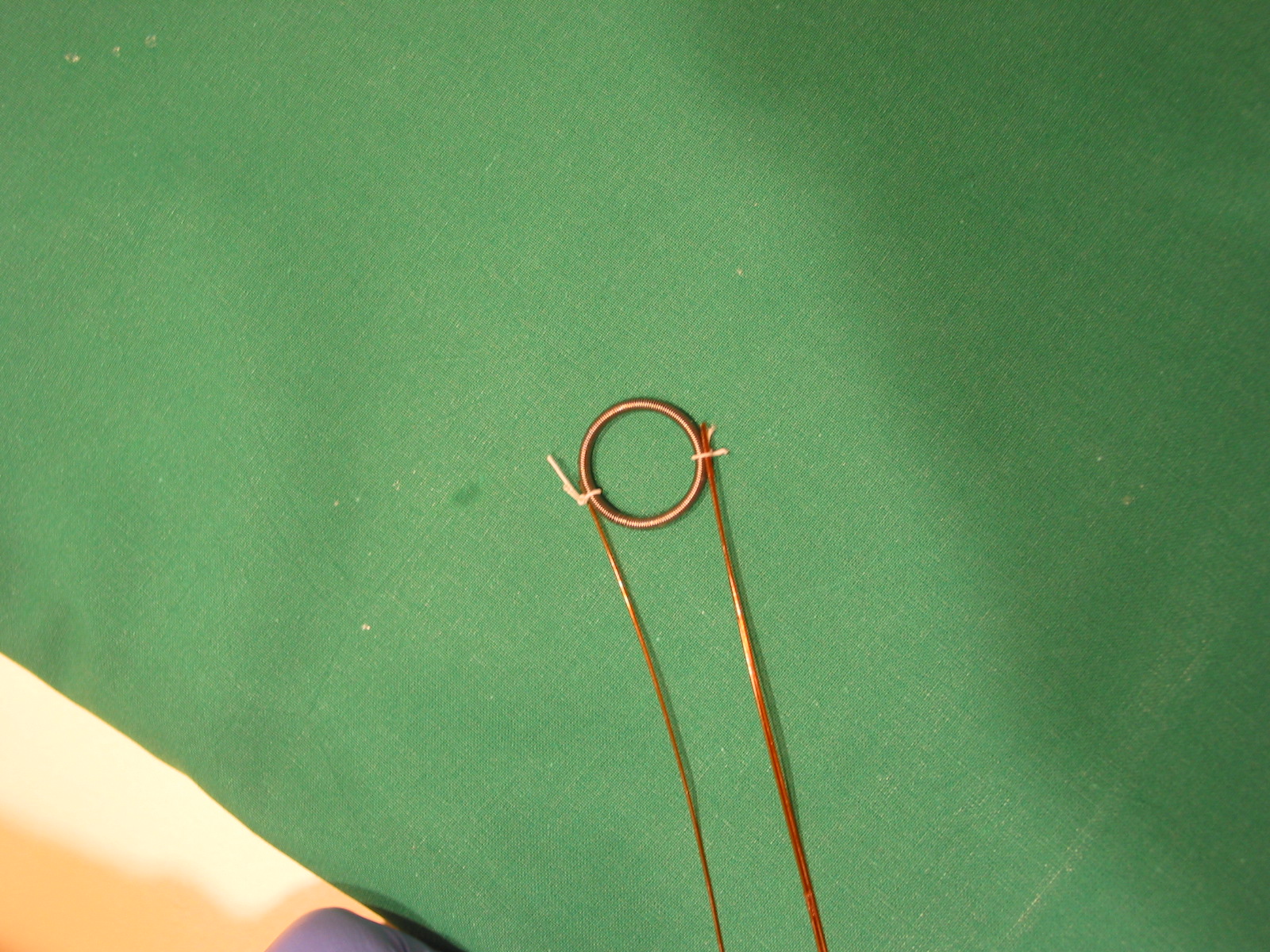

Supplement: S1 File — (ZIP) [file pone.0204220.s001.zip › rf_heating/S36.JPG]

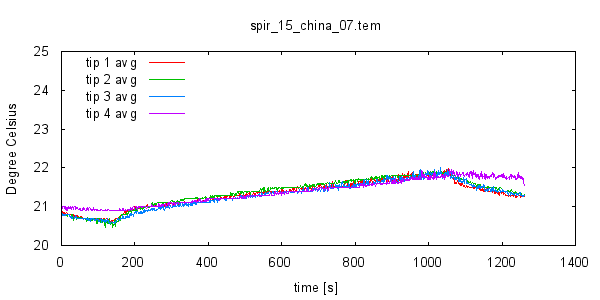

Supplement: S1 File — (ZIP) [file pone.0204220.s001.zip › rf_heating/spir_15_china_07.gif]

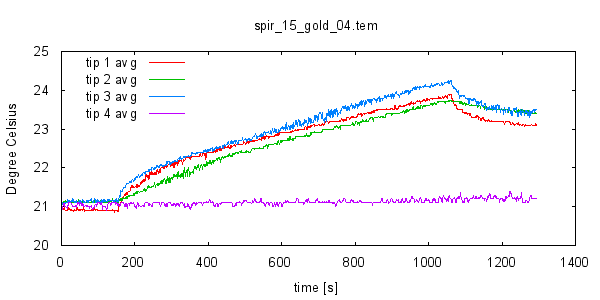

Supplement: S1 File — (ZIP) [file pone.0204220.s001.zip › rf_heating/spir_15_gold_04.gif]

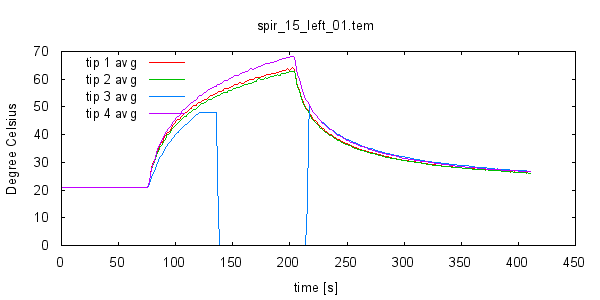

Supplement: S1 File — (ZIP) [file pone.0204220.s001.zip › rf_heating/spir_15_left_01.gif]

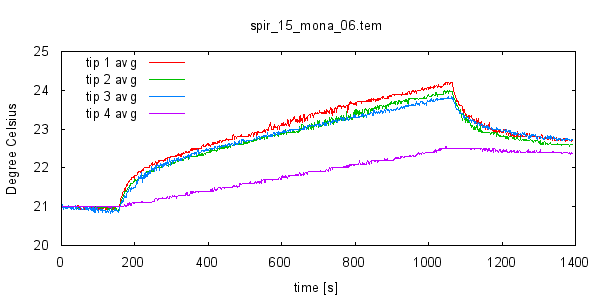

Supplement: S1 File — (ZIP) [file pone.0204220.s001.zip › rf_heating/spir_15_mona_06.gif]

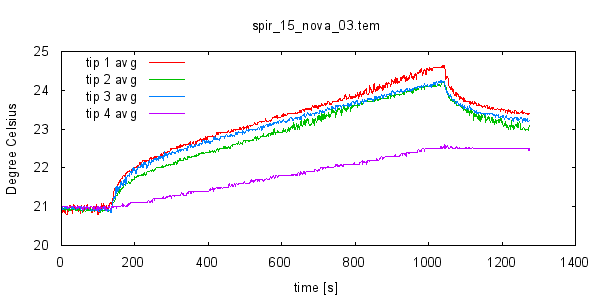

Supplement: S1 File — (ZIP) [file pone.0204220.s001.zip › rf_heating/spir_15_nova_03.gif]

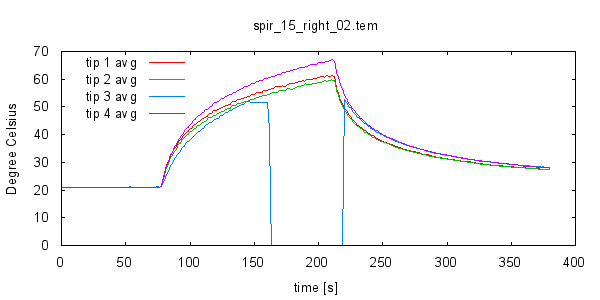

Supplement: S1 File — (ZIP) [file pone.0204220.s001.zip › rf_heating/spir_15_right_02.gif]
